# Accounting for selection and correlation in the analysis of two-stage genome-wide association studies

**DOI:** 10.1093/biostatistics/kxw012

**Published:** 2016-03-18

**Authors:** David S. Robertson, A. Toby Prevost, Jack Bowden

**Affiliations:** MRC Biostatistics Unit, IPH Forvie Site, Robinson Way, Cambridge CB2 0SR, UK; Imperial College London, 1st Floor, Stadium House, 68 Wood Lane, London W12 7RH, UK; MRC Integrative Epidemiology Unit, University of Bristol, Oakfield House, Bristol BS8 2BN, UK and MRC Biostatistics Unit, IPH Forvie Site, Robinson Way, Cambridge CB2 0SR, UK

**Keywords:** Correlated outcomes, Genome-wide scan, Selection bias, Two-stage sample, Uniformly minimum variance conditionally unbiased estimator

## Abstract

The problem of selection bias has long been recognized in the analysis of two-stage trials, where promising candidates are selected in stage 1 for confirmatory analysis in stage 2. To efficiently correct for bias, uniformly minimum variance conditionally unbiased estimators (UMVCUEs) have been proposed for a wide variety of trial settings, but where the population parameter estimates are assumed to be independent. We relax this assumption and derive the UMVCUE in the multivariate normal setting with an arbitrary known covariance structure. One area of application is the estimation of odds ratios (ORs) when combining a genome-wide scan with a replication study. Our framework explicitly accounts for correlated single nucleotide polymorphisms, as might occur due to linkage disequilibrium. We illustrate our approach on the measurement of the association between 11 genetic variants and the risk of Crohn's disease, as reported in Parkes *and others* (2007. Sequence variants in the autophagy gene IRGM and multiple other replicating loci contribute to Crohn's disease susceptibility. *Nat. Gen.* **39**(7), 830–832.), and show that the estimated ORs can vary substantially if both selection and correlation are taken into account.

## 1. Introduction

The two-stage “learn and confirm” strategy is widely implemented across biomedical research. For example, in the sphere of randomized clinical trials, the vast majority of adaptive trial designs follow a two-stage strategy, where design adaptations are made at the first interim analysis, with the trial then proceeding under a fixed design until its completion. See Bauer *and others* (2016), Bretz *and others* (2009), and Posch *and others* (2005) for a thorough review of the literature.

A key application has been in two-stage adaptive seamless trials incorporating mid-trial treatment selection (Bowden and Glimm, 2014; Kimani *and others*, 2013; Sampson and Sill, 2005). Typically, multiple experimental treatments are simultaneously compared with a control in stage 1, and the most promising treatment is selected for confirmatory analysis in stage 2.

In biomedical science more generally, there is an increasing focus on the identification of genetic markers predictive of phenotypic variation, in order to better understand disease etiology and to target future drug development. The design of choice is the genome-wide association study (GWAS), in which upwards of one million single nucleotide polymorphisms (SNPs) can be tested simultaneously for their association with a given phenotype. Those SNPs achieving “genome-wide” significance (typically defined as a $p$-value of $<10^{-8}$) are then validated in a replication study (Ioannidis *and others*, 2009).

A whole variety of formal two-stage design procedures have been proposed in this setting; see, for example, Satagopan and Elston (2003), Satagopan *and others* (2004, 2002), Thomas *and others* (2004), with an overview provided in Thomas *and others* (2009). In practice, the two-stage GWAS approach has been successfully applied to many disease areas, including sclerosis (Chiò *and others*, 2009), epilepsy (Guo *and others*, 2012), and breast cancer (Sapkota *and others*, 2012).

However, ranking and selecting candidates in a two-stage trial can induce bias in the final estimates of the parameter of interest. Indeed, as a general rule, the more aggressive the selection, the worse is the bias (Bauer *and others*, 2010). Specifically, a candidate has to perform “well” in stage 1 in order to proceed to stage 2, which often leads to overly optimistic estimates, or the so-called “winner's curse”. When there is heavy selection from many candidates of roughly equivalent parameters, then the selected candidate is typically based on chance variability rather than true superiority (Sill and Sampson, 2007). All this calls into question the generalizability of the trial's findings to future patients.

The need to correct for this selection bias in the analysis of two-stage trials has long been recognized. An efficient and unbiased estimator can be obtained through the technique of Rao–Blackwellization. This involves taking the unbiased stage 2 data and conditioning on a complete, sufficient statistic for the parameter in question. By the Lehmann–Scheffé theorem, this gives the uniformly minimum variance conditionally unbiased estimator (UMVCUE).

This two-stage framework was introduced by Cohen and Sackrowitz (1989), who derived the UMVCUE for the selected mean, where the stage 1 populations are independent and normally distributed. The method lends itself naturally to estimation in two-stage adaptive seamless phase II/III clinical trials (Bowden and Glimm, 2008; Kimani *and others*, 2013), with equivalent work carried out in biomarker research (Pepe *and others*, 2009; Koopmeiners *and others*, 2012; Robertson *and others*, 2015). In the GWAS setting, Bowden and Dudbridge (2009) extended the Cohen and Sackrowitz method to derive the UMVCUE for the SNP-disease log odds ratios (ORs), where either a one-sided or two-sided $p$-value test is used for ranking the SNPs.

A crucial assumption in all of the above analyses is that the stage 1 population parameter estimates are *independent* random variables. However, this may not be a reasonable assumption to make. For example, in the GWAS setting, SNPs in the same chromosomal region may be in linkage disequilibrium (LD).

The aim of this paper is to present general theory to enable the calculation of UMVCUEs for each setting that explicitly accounts for correlation. We achieve this by deriving the UMVCUE in the multivariate normal setting with an arbitrary known covariance structure. We illustrate the utility of the new theory using an application in the GWAS setting.

Since the focus of this paper *is* on point estimation, our primary criterion for comparing the performance of different estimators is the magnitude of the bias. Of course, bias is not the only criterion with which to judge an estimator, especially considering the bias–variance trade-off. However, it is important to quantify the bias of an estimator, not least due to regulatory concerns. As a secondary criterion, we also compare the mean squared error (MSE) of the estimators, which takes into account the variance. In practice, the relative importance attached to the criterion of bias and MSE will vary from setting to setting.

In Section 2, we describe the general model framework and derive the form of the multivariate normal UMVCUE. We then present a short simulation study in Section 3 for the bivariate normal case. We extend our framework to estimating ORs in genome-wide scans in Section 4, and apply the resulting UMVCUE to data reported in Parkes *and others* (2007). We conclude with a discussion in Section 5.

## 2. General framework for the UMVCUE

### 2.1. Model description

Consider the following two-stage trial design. Suppose that we have $K$ correlated continuous stage 1 parameter estimates, defined by ${\boldsymbol X} = (X_1, \ldots , X_K)$, for the candidate treatments, biomarkers or genetic variants $C_1, \ldots , C_K$. Let ${\boldsymbol X}$ follow a multivariate normal distribution, defined by ${\boldsymbol X} \sim N(\boldsymbol {\mu }, V)$, where $\boldsymbol {\mu }$ is the vector of (unknown) means and $V = (V_{ij}), i,j \in \{1, \ldots , K \}$ is the covariance matrix. We assume that $V$ is known and is positive definite. For notational convenience, we let $V_{ii} = \sigma _i^2$ for $i = 1, \ldots , K$.

We denote the ordered stage 1 estimates as $X_{(i)}$, where the ordering is simply by effect size: $X_{(1)} > X_{(2)} > \cdots > X_{(K)}$. We consider other ordering mechanisms in due course. Let $Y_i$ be the stage 2 estimate of the $i$th ranked candidate, and so $Y_i \sim N(\mu _{(i)}, \tau _i^2)$. At the end of stage 2, the aim is to efficiently estimate $\mu _{(j)}$ for some $j \in \{ 1, \ldots , K \}$.

The MLE for $\mu _{(j)}$ is a weighted average of the first- and second-stage data:

\[ \hat{\mu}_{(j)} = \frac{\tau_j^2 X_{(j)} + \sigma_{(j)}^2 Y_j}{\sigma_{(j)}^2 + \tau_j^2}. \]

This estimator may be biased, because it does not take into account the first-stage selection procedures or the correlation. An unbiased estimator can easily be found by just using the stage 2 data $Y_j$. However, this estimator suffers from lower precision since we are neglecting the stage 1 data. Hence we look for an unbiased estimator that uses data from both stages.

### 2.2. Calculating the UMVCUE

Let $Q$ be the event $\{{\boldsymbol X}: X_1 > X_2 > \cdots > X_K \}$, which implies that $X_{(i)} = X_i$ for $i = 1, \ldots , K$. Without loss of generality, we condition on $Q$ for the remainder of the derivation of the UMVCUE.

To start with, consider estimating the mean of the highest ranked population, $\mu _1$. For notational convenience, we let $Y_1 = Y$ and $\tau _1 = \tau $. In Section 1 of the supplementary materials (available at *Biostatistics* online), we prove the following theorem for the complete and sufficient statistic for $\boldsymbol {\mu } = (\mu _1, \ldots , \mu _K)$.

**Theorem 2.1.**

The statistic ${\boldsymbol Z} = (Z_1, \ldots , Z_K)$ is sufficient and complete for $\boldsymbol {\mu } = (\mu _1, \ldots , \mu _K)$, where $Z_i = X_i + ({V_{1i}}/{\tau ^2}) Y$ for $i = 1, \ldots , K$.

Then, by the Lehmann–Scheffé theorem, $\hat {U} = E ( Y \mid {\boldsymbol Z} = {\boldsymbol z}, Q )$ is the UMVCUE for $\mu _1$ under $Q$. Hence we have the following result, as proved in Section 2 of the supplementary materials (available at *Biostatistics* online).

**Theorem 2.2.**

The UMVCUE for $\mu _1$ given $Q$ is

(2.1) \begin{equation*} \hat{U} = \frac{\tau^2 Z_1}{\sigma_1^2 + \tau^2} - \frac{\tau^2}{\sqrt{\sigma_1^2 + \tau^2}} \frac{\phi(W_1) - \phi(W_2)}{\Phi(W_1) - \Phi(W_2)}, \end{equation*}

where

\begin{align*} W_i &= \frac{k_i \sqrt{\sigma_1^2 + \tau^2}}{\tau^2} - \frac{Z_1}{\sqrt{\sigma_1^2 + \tau^2}} \quad \hbox{for } i = 1,2,\\ k_1 &= \min(A_1), \quad k_2 = \max(A_2), \\ A_1 &= \left\{\frac{\tau^2 (Z_j - Z_{j+1})}{V_{1j} - V_{1,j+1}} : V_{1j} > V_{1,j+1}; \ j = 1, \ldots, K-1 \right\}, \\ A_2 &= \left\{\frac{\tau^2 (Z_j - Z_{j+1})}{V_{1j} - V_{1,j+1}} : V_{1j} < V_{1,j+1}; \ j = 1, \ldots, K-1 \right\} \end{align*}

and we define $\min \{\emptyset \} = +\infty $ and $\max \{\emptyset \} = -\infty $.

**Remark.**

In the independent normal setting, Cohen and Sackrowitz (1989) note that all dependency on observations $X_3, X_4, \ldots , X_K$ vanished in the expression for the UMVCUE—which they interpreted as a negative result when $K>2$. However, we see in Equation (2.1) that accounting for correlation means that all of the observations can contribute to the sets $A_1$ and $A_2$ and be implicitly used in the estimator. In fact, any of the observations $X_3, X_4, \ldots , X_K$ can be used *explicitly*, depending on which feature in $\min (A_1)$ and $\max (A_2)$.

**Remark 2.**

It is often the case that the stage 1 estimates are asymptotically normal rather than precisely normal. For example, log ORs have an asymptotic normal distribution. In these settings, our theorems hold asymptotically.

Suppose that, instead of only selecting the single top-ranking treatment or biomarker for continuation to stage 2, we take forward the top $K$ from a larger group of $K'$. For example, in the GWAS setting, typically $K' \approx 500\,000$ and $K < 100$. We now require the UMVCUE for the $j$th best candidate out of $K$ and the following corollary covers this case.

**Corollary 2.3.**

For a given value of $j \in \{1, \ldots , K\}$, the statistic ${{\boldsymbol Z}_j} = (Z_{1j}, \ldots , Z_{Kj})$ is sufficient and complete for $\boldsymbol {\mu } = (\mu _1, \ldots , \mu _K)$, where

(2.2) \begin{equation*} Z_{ij} = X_i + \frac{V_{ij}}{\tau_j^2} Y_j \end{equation*}

for $i = 1, \ldots , K$.

Also, the UMVCUE for $\mu _j$ given $Q$ is

(2.3) \begin{equation*} \hat{U}_j = \frac{\tau_j^2 Z_{jj}}{\sigma_j^2 + \tau_j^2} - \frac{\tau_j^2}{\sqrt{\sigma_j^2 + \tau_j^2}} \frac{\phi(W_1) - \phi(W_2)}{\Phi(W_1) - \Phi(W_2)}, \end{equation*}

where

\begin{align*} W_i &= \frac{k_i \sqrt{\sigma_j^2 + \tau_j^2}}{\tau_j^2} - \frac{Z_{jj}}{\sqrt{\sigma_j^2 + \tau_j^2}} \quad \hbox{for } i = 1,2,\\ k_1 &= \min(A_1), \quad k_2 = \max(A_2), \\ A_1 &= \left\{ \frac{\tau_j^2 (Z_{ij} - Z_{i+1,j})}{V_{ij} - V_{i+1,j}} : V_{ij} > V_{i+1,j}; \ i = 1, \ldots, K-1 \right\}, \\ A_2 &= \left\{ \frac{\tau_j^2 (Z_{ij} - Z_{i+1,j})}{V_{ij} - V_{i+1,j}} : V_{ij} < V_{i+1,j}; \ i = 1, \ldots, K-1 \right\} \end{align*}

and we define $\min \{\emptyset \} = +\infty $ and $\max \{\emptyset \} = -\infty $.

**Remark.**

As a simple check of our methodological development's consistency with previous results, if we set the off-diagonal terms of the covariance matrix $V$ equal to zero, then we recover the independent normal setting. The UMVCUE was derived in this special case by Bowden and Glimm (2008). We compare our results in Section 3 of the supplementary materials (available at *Biostatistics* online) and show that they are the same.

In Section 4, we show the form of the UMVCUE when ranking by $p$-value, and subsequently show how to construct bootstrapped confidence intervals (CIs) in this setting.

## 3. Simulation study: bivariate normal case

### 3.1. Bivariate normal UMVCUE

We now consider the UMVCUE derived in the bivariate normal setting, where $K = 2$. Suppose we have two correlated continuous stage 1 outcome measures, defined by ${\boldsymbol X} = (X_1, X_2)$, for the candidates $C_1$ and $C_2$. We let ${\boldsymbol X}$ follow a bivariate normal distribution, with

\[ \begin{pmatrix} X_1 \\ X_2 \end{pmatrix} \sim N \left( \begin{pmatrix} \mu_1 \\ \mu_2 \end{pmatrix} , \begin{pmatrix} \sigma_1^2 & \rho \sigma_1 \sigma_2\\ \rho \sigma_1 \sigma_2 & \sigma_2^{2} \end{pmatrix}\right), \]

where $\rho $ is the correlation coefficient between $X_1$ and $X_2$, and we assume $\rho \neq \pm 1$.

From Equations (2.1), the statistic $(Z_1, Z_2)$ is sufficient for $(\mu _1, \mu _2)$, where

\[ Z_1 = X_1 + \frac{\sigma_1^2}{\tau^2} Y, \quad Z_2 = X_2 + \frac{\rho \sigma_1 \sigma_2}{\tau^2} Y. \]

The UMVCUE depends on whether $V_{11} > V_{12} \iff {\sigma _1}/{\sigma _2} > \rho $. Since $ {\tau ^2 (Z_1 - Z_2)}/({V_{11} - V_{12}}) = {\tau ^2 (Z_1 - Z_2)}/({\sigma _1^2 - \rho \sigma _1 \sigma _2}),$ if follows that, from Equation (2.1), the UMVCUE for $\mu _1$ given $Q$ is

(3.1) \begin{equation*} \hat{U} = \begin{cases} \dfrac{\tau^2 Z_1}{\sigma_1^2 + \tau^2} - \dfrac{\tau^2}{\sqrt{\sigma_1^2 + \tau^2}} \dfrac{\phi(W)}{\Phi(W)} & \hbox{if } \dfrac{\sigma_1}{\sigma_2} > \rho, \\ \dfrac{\tau^2 Z_1}{\sigma_1^2 + \tau^2} + \dfrac{\tau^2}{\sqrt{\sigma_1^2 + \tau^2}} \dfrac{\phi(W)}{\Phi(-W)} & \hbox{if } \frac{\sigma_1}{\sigma_2} < \rho, \\ \dfrac{\tau^2 Z_1}{\sigma_1^2 + \tau^2} & \hbox{if } \dfrac{\sigma_1}{\sigma_2} = \rho, \end{cases} \end{equation*}

where

\[ W = \frac{(Z_1 - Z_2) \sqrt{\sigma_1^2 + \tau^2}}{\sigma_1^2 - \rho \sigma_1 \sigma_2} - \frac{Z_1}{\sqrt{\sigma_1^2 + \tau^2}}. \]

**Remark.**

Note that if ${\sigma _1}/{\sigma _2} = \rho $, then the UMVCUE is in fact equal to the MLE $\hat {\mu }_1$.

### 3.2. Simulation results

In this subsection, we compare various estimators for the largest mean $\mu _1$ in the bivariate normal setting:

$Y = {\rm the}$ observed stage 2 data for the highest ranked population.
$\hat {\mu }_1 = {\rm the}$ MLE for $\mu _{1}$, which ignores selection and correlation.
$\hat {U}_{{\rm B}} = {\rm the}$ UMVCUE derived by Bowden and Glimm (2008), which accounts for selection but ignores correlation. Note that this is equivalent to $\hat {U}$ when we set $\rho = 0$.
$\hat {U} = {\rm our}$ UMVCUE given in Equation (3.1), which accounts for selection and correlation.

To evaluate the performance of a generic estimator for $\mu _{1}$, say $\mu _{1}^{*}$, we calculate the bias and MSE as defined in Posch *and others* (2005), Sill and Sampson (2007), and Bowden and Glimm (2008):

\begin{align*} b_{{\rm sel}}(\mu_{1}^{*}) & = \sum_{i = 1}^{K} E [\mu_{1}^{*} - \mu_i \mid X_{1} = X_i] P ( X_{1} = X_i), \\ \hbox{MSE}_{{\rm sel}}(\mu_{1}^{*}) & = \sum_{i = 1}^{K} E [(\mu_{1}^{*} - \mu_i)^2 \mid X_{1} = X_i]P (X_{1} = X_i). \end{align*}

In our simulation study, we set $\sigma _1 = 0.05$ and $\sigma _2 = 0.1$ and then consider four simple scenarios (i)–(iv) with varying values of the true means $\boldsymbol {\mu } = (\mu _1, \mu _2)$ and stage 2 variance $\tau ^2$:

\begin{align*} & \hbox{(i)} \ \boldsymbol{\mu} = (0.1, 0.1), \tau = 0.05, \quad \hbox{(ii)} \ \boldsymbol{\mu} = (0.1,0.3), \tau = 0.05, \\ & \hbox{(iii)} \ \boldsymbol{\mu} = (0.1,0.1), \tau = \sigma_{1},\quad \hbox{(iv)} \ \boldsymbol{\mu} = (0.1,0.3), \tau = \sigma_{1}. \end{align*}

Note that, for scenarios (i) and (ii), $\hbox {MSE}_{{\rm sel}}(Y) = 0.05^2$. For scenarios (iii) and (iv), the exact MSE of $Y$ is as follows:

\begin{align*} \hbox{MSE}_{{\rm sel}}(Y) & = E [(Y - \mu_1)^2 \mid X_1 > X_2 ] P ( X_1 > X_2 ) + E [(Y - \mu_2)^2 \mid X_1 < X_2]P (X_1 < X_2)\\ & = \sigma_1^2 \Phi \left( \frac{\mu_1-\mu_2}{\sqrt{\sigma_1^2+\sigma_2^2-2\rho\sigma_1\sigma_2}} \right) + \sigma_2^2 \left[ 1 - \Phi \left( \frac{\mu_1-\mu_2}{\sqrt{\sigma_1^2+\sigma_2^2-2\rho\sigma_1\sigma_2}} \right) \right]. \end{align*}

**Fig. 1.**
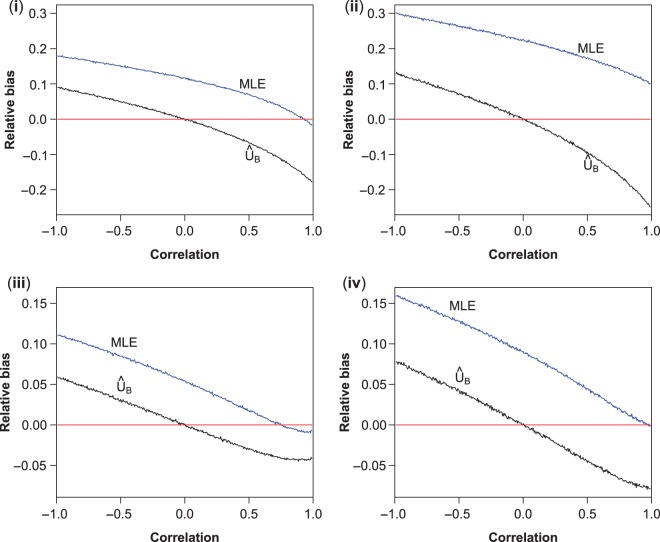
Mean relative bias of the MLE ($\hat {\mu }_1$, blue), and the UMVCUE ($\hat {U}_B$, black) that ignores the correlation. Plots show the mean of 100 000 simulations for each value of $\rho $. The horizontal line shows where the bias equals zero.

Figure 1 shows the mean relative bias (defined as ${b_{{\rm sel}}}/{\mu _1}$) of the MLE $(\hat {\mu }_1)$ and the UMVCUE $(\hat {U}_B)$ (which ignores correlation) as a function of $\rho $ for the four scenarios. Since the mean bias of the stage 2 data $(Y)$ and the UMVCUE $(\hat {U})$ are virtually identical to zero for all values of $\rho $ (by definition), we do not show the simulation results here.

The plots show that the bias of both estimators is at a maximum for $\rho $ close to $-1$. The MLE is positively biased for $\rho < 0.5$, and in scenarios (ii) is in fact substantially biased for all values of $\rho $. We see that $\hat {U}_B$ is positively biased for $\rho < 0$, unbiased when $\rho = 0$ (as would be expected), and negatively biased for $\rho > 0$. The bias of both estimators is worse when $\tau = \sigma _1$ compared with $\tau = 0.05$.

**Fig. 2.**
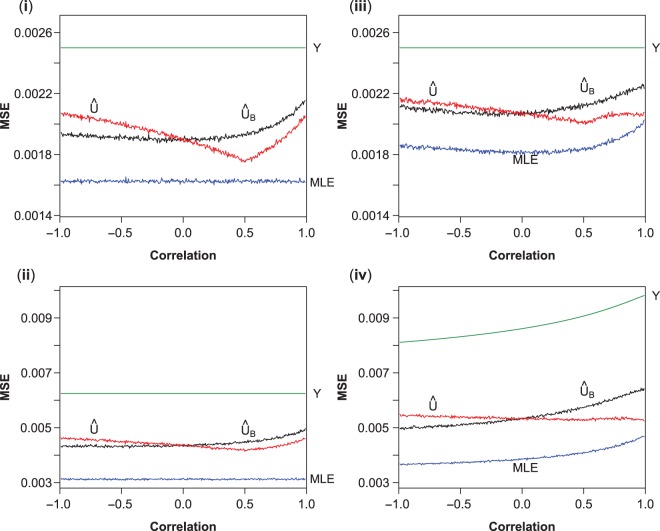
MSE of the MLE (blue), $Y$ (green), $\hat {U}_B$ (black), and $\hat {U}$ (red). Plots show the mean of 100 000 simulations for each value of $\rho $, except for $Y$, which is the theoretical value of the MSE.

Figure 2 shows the MSE of the estimators $(\hat {\mu }_1, Y, \hat {U}_B, \hat {U})$ for the four scenarios. As would be expected, the MLE always has the lowest MSE, while $Y$ always has the highest. We also see that there is a substantial reduction in the MSE when using the UMVCUEs $\hat {U}$ or $\hat {U}_B$.

In scenarios (i)–(iii), the MSE of $\hat {U}$ decreases as $\rho $ approaches 0.5, and then increases again. This is to be expected given Equation (3.1), which shows that $\hat {U}$ reduces down to the MLE when $\sigma _{1} = \rho \sigma _{2}$. In scenarios (iii) and (iv), we also see that, for $\rho $ close to 1, the MSE of $\hat {U}$ and the MLE are very close.

In all of the scenarios, the MSE of $\hat {U}$ is greater than that for $\hat {U}_B$ when $\rho < 0$, equal when $\rho = 0$ (as would be expected), and less than that for $\hat {U}_B$ when $\rho > 0$. However, these differences in the MSE are much less than the differences between the MSE of $Y$ and either $\hat {U}$ or $\hat {U}_B$. In conclusion, there is little or no advantage in terms of MSE when using the estimator $\hat {U}_B$ as opposed to our UMVCUE $\hat {U}$, which properly accounts for correlation.

## 4. Application to the Parkes and others' GWAS data

We now apply our methodology to data from the genome-wide association scan for Crohn's disease published in 2007 by the Wellcome Trust Case Control Consortium (2007) (WTCCC). There were 1748 cases for the disease, and 2938 controls in this cohort, which identified 12 SNPs associated with disease status at genome-wide significance.

A replication study was then reported by Parkes *and others* (2007) in a follow-up cohort of 1182 cases and 2024 controls, in which 12 SNPs were successfully replicated in this study. Following Bowden and Dudbridge (2009), we exclude one (rs6887695) from our illustration (as its significance in the WTCCC scan was severely reduced after data cleaning).

Table 1 shows the estimated ORs for stages 1 and 2 (i.e. the WTCCC data and the replication data), as well as the overall MLE. This is then followed by two previously described estimators that combine the data from the two stages in a way that attempts to correct for bias. The first estimator (denoted by ZP) is the corrected MLE of Zhong and Prentice (2008). The second (denoted by $\hat {U}_B$) is the estimator considered in Bowden and Dudbridge (2009), who calculated the UMVCUE assuming that all of the log ORs for the SNPs are uncorrelated.

**Table 1.** The ORs ($95\%$ CIs) of 11 SNPs estimated from allele frequencies reported by Parkes *and others* (2007) in an initial scan (stage 1) and replication study (stage 2). SNPs are ranked in order of stage 1 statistical significance, with those on the same chromosomal region highlighted in bold

Chr	SNP	Stage 1 OR	Stage 2 OR	MLE	ZP	$\hat {U}_{\hbox {B}}$
**5p13**	**rs17234657**	**1.55 (1.38, 1.74)**	**1.16 (1.00, 1.35)**	**1.39**	**1.39**	**1.16**
**5p13**	**rs9292777**	**1.38 (1.26, 1.51)**	**1.34 (1.20, 1.50)**	**1.37**	**1.37**	**1.39**
10q24	rs10883365	1.27 (1.17, 1.38)	1.18 (1.05, 1.32)	1.24	1.24	1.16
18p11	rs2542151	1.35 (1.21, 1.50)	1.15 (1.00, 1.32)	1.27	1.25	1.15
**5q33**	**rs13361189**	**1.51 (1.30, 1.76)**	**1.38 (1.15, 1.66)**	**1.46**	**1.45**	**1.40**
3p21	rs9858542	1.26 (1.15, 1.38)	1.17 (1.04, 1.31)	1.22	1.21	1.17
**5q33**	**rs4958847**	**1.35 (1.20, 1.53)**	**1.36 (1.17, 1.59)**	**1.36**	**1.35**	**1.35**
5q23	rs10077785	1.29 (1.16, 1.43)	1.19 (1.05, 1.36)	1.25	1.22	1.19
1q24	rs12035082	1.22 (1.12, 1.33)	1.14 (1.02, 1.27)	1.19	1.17	1.15
21q22	rs2836754	1.22 (1.12, 1.33)	1.15 (1.03, 1.28)	1.19	1.16	1.16
1q31	rs10801047	1.38 (1.18, 1.61)	1.47 (1.22, 1.76)	1.42	1.39	1.44

As the bold entries in Table 1 indicate, two pairs of SNPs are on the same chromosomal region. The first- and second-ranked SNPs (rs17234657 and rs9292777) are both on chromosomal region 5p13, while the fifth- and seventh-ranked SNPs (rs13361189 and rs4958847) are both on chromosomal region 5q33.

It can be safely assumed that SNPs from distinct genomic regions are in linkage equilibrium. However, in an effort to assess the potential correlation between SNPs in the same genomic region, we used the data from 498 Europeans in the 1000 genomes project (1000 Genomes Project Consortium, 2012). The empirical correlation between the SNPs (encoded as 0,1,2 to indicate the number of risk increasing alleles) on chromosomal regions 5p13 and 5q33 were $-0.28$ and $0.78,$ respectively.

Although the populations considered by Parkes *and others* (2007) and the 1000 genomes project are different, these correlations do suggest LD exists between these SNPs. Hence a natural question to ask is how the OR estimates are affected by correlation. To do so, we first need to extend our framework to account for ranking by $p$-value.

### 4.1. Calculating the UMVCUE

Consider a two-stage design with a genome-wide association scan (stage 1) followed by a replication study (stage 2). Only those SNPs that meet selection criteria (see below) in stage 1 continue on to stage 2. A common approach in the genome-wide setting is to rank the SNPs according to the statistical significance of the effects, with a $p$-value threshold $p_{\hbox {crit}}$. We assume that $K$ of the SNPs pass $p_{\hbox {crit}}$ and are then ranked.

For a one-sided test, we condition (WLOG) on the event

\[ Q_1 = \left\{{\boldsymbol X}: \frac{X_1}{\sigma_1} \geq \frac{X_2}{\sigma_2} \geq \cdots \geq \frac{X_K}{\sigma_K} \geq \Phi^{-1}(1 - p_{\hbox{crit}}) \right\}. \]

The UMVCUE in this case is given in Section 4 the supplementary materials (available at *Biostatistics* online).

For a two-sided test, we instead condition (WLOG) on the event

\[ Q_2 = \left\{{\boldsymbol X}: \frac{|X_1|}{\sigma_1} \geq \frac{|X_2|}{\sigma_2} \geq \cdots \geq \frac{|X_K|}{\sigma_K} \geq \Phi^{-1}(1 - p_{\hbox{crit}}/2) \right\}. \]

Then the UMVCUE for $\mu _j$ is as follows, with the proof found in Section 5 of the supplementary materials (available at *Biostatistics* online):

**Theorem 4.1.**

The UMVCUE for $\mu _j$ given $Q_2$ is

(4.1) \begin{equation*} \hat{U}_j = \frac{\tau_j^2 Z_{jj}}{\sigma_j^2 + \tau_j^2} - \frac{\tau_j^2}{\sqrt{\sigma_j^2 + \tau_j^2}} \frac{\sum_{i = 1}^M \phi(W_{1i}) - \phi(W_{2i})}{\sum_{i = 1}^M \Phi(W_{1i}) - \Phi(W_{2i})}, \end{equation*}

where

\begin{align*} W_{1i} &= \frac{b_i \sqrt{\sigma_j^2 + \tau_j^2}}{\tau_j^2} - \frac{Z_{jj}}{\sqrt{\sigma_j^2 + \tau_j^2}}, \quad W_{2i} = \frac{a_i \sqrt{\sigma_j^2 + \tau_j^2}}{\tau_j^2} - \frac{Z_{jj}}{\sqrt{\sigma_j^2 + \tau_j^2}}, \\ \bigcup_{i=1}^M [a_i, b_i] &= \left(\bigcap_{i=1}^{K-1} (A_{1i} \cap A_{2i}) \cup (A_{3i} \cap A_{4i})\right) \cap (A_5 \cup A_6), \\ A_{1i} &= \{Y : (\sigma_i V_{i+1,j} - \sigma_{i+1} V_{ij})Y \geq \tau_j^2 (\sigma_i Z_{i+1,j} - \sigma_{i+1} Z_{ij})\}, \\ A_{2i} &= \{Y : (\sigma_i V_{i+1,j} + \sigma_{i+1} V_{ij})Y \leq \tau_j^2 (\sigma_{i+1} Z_{ij} + \sigma_i Z_{i+1,j})\}, \\ A_{3i} &= \{Y : (\sigma_i V_{i+1,j} + \sigma_{i+1} V_{ij})Y \geq \tau_j^2 (\sigma_{i+1} Z_{ij} + \sigma_i Z_{i+1,j})\}, \\ A_{4i} &= \{Y : (\sigma_i V_{i+1,j} - \sigma_{i+1} V_{ij})Y \leq \tau_j^2 (\sigma_i Z_{i+1,j} - \sigma_{i+1} Z_{ij})\}, \\ A_5 &= \{ Y : V_{Kj} Y \leq \tau_j^2 [Z_K - \sigma_{K} \Phi^{-1}(1 - p_{\hbox{crit}}/2) ]\}, \\ A_6 &= \{ Y : V_{Kj} Y \geq \tau_j^2 [Z_K + \sigma_{K} \Phi^{-1}(1 - p_{\hbox{crit}}/2)]\} \end{align*}

and we define $\min \{\emptyset \} = +\infty $ and $\max \{\emptyset \} = -\infty $.

**Remark.**

The introduction of the variable $M$ above is for notational convenience. In particular, we re-express the intersection of sets $(\bigcap _{i=1}^{K-1} (A_{1i} \cap A_{2i}) \cup (A_{3i} \cap A_{4i})) \cap (A_5 \cup A_6)$ as the disjoint union of $M$ closed intervals on the real line $[a_1, b_1], \ldots , [a_M, b_M]$, where $M \geq 1$. Note that possibly $a_1 = -\infty $ and/or $b_M = \infty $.

For either test, if all the off-diagonal terms of the covariance matrix are equal to zero, then we recover the independent normal setting as in Bowden and Dudbridge (2009). In Section 6 of the supplementary materials (available at *Biostatistics* online), we compare our estimators and show they are the same.

### 4.2. Results

Rather than assuming we know $\rho $, we take each pair of SNPs that are on the same chromosomal region and calculate the UMVCUEs as the correlation between the log ORs varies between $\pm 1$. More explicitly, given a pair of SNPs on the same chromosomal region which have stage 1 ranks $j_1$ and $j_2,$ respectively, then we assume that the log ORs $X_{j_1}$ and $X_{j_2}$ follow a bivariate normal distribution, with correlation coefficient $\rho $

\[ \begin{pmatrix} X_{j_1} \\ X_{j_2} \end{pmatrix} \sim N \left( \begin{pmatrix} \mu_{j_1} \\ \mu_{j_2} \end{pmatrix}\!, \begin{pmatrix} \sigma_{j_1}^2 & \rho \sigma_{j_1} \sigma_{j_2}\\ \rho \sigma_{j_1} \sigma_{j_2} & \sigma_{j_2}^{2} \end{pmatrix}\right). \]

**Fig. 3.**
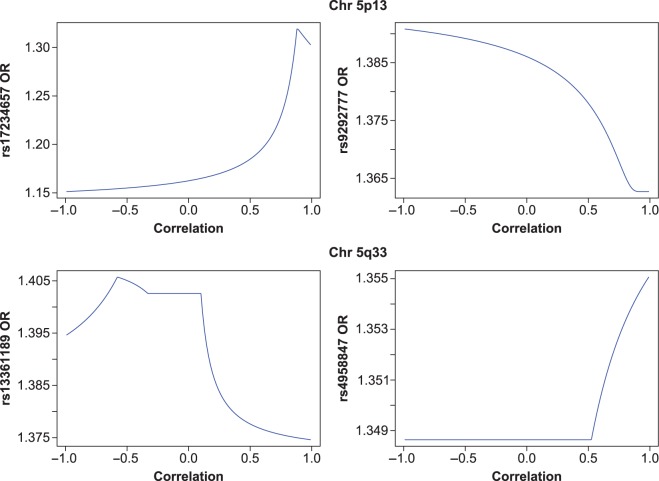
The UMVCUEs for the ORs of the SNPs on chromosomal region 5p13 (rs17234657 and rs9292777) and 5q33 (rs13361189 and rs4958847) as a function of the correlation coefficient.

Starting with the SNPs on chromosomal region 5p13, the upper half of Figure 3 shows the UMVCUEs for the ORs as a function of the correlation coefficient $\rho $. The first plot for SNP rs17234657 shows that the UMVCUE can vary substantially if there is positive correlation between the SNPs. As $\rho $ increases from 0 to 0.88, the UMVCUE increases from 1.16 to 1.32, with a particularly rapid increase for $\rho $ greater than about 0.5. For $\rho > 0.88$ we actually see a (small) decrease in the UMVCUE. In contrast, for negative values of $\rho $, the UMVCUE is hardly affected.

The second plot for SNP rs9292777 shows that the UMVCUE decreases as $\rho $ increases, but in absolute terms correlation has little effect. The main reason for this is because the stage 1 and stage 2 estimates for the OR were very similar (1.38 and 1.34, respectively), so we would not expect the UMVCUE to be dramatically different regardless of any correlation.

Performing the analysis for the SNPs on chromosomal region 5q33 gives the results displayed in the lower half of Figure 3. Looking at the first plot for SNP rs13361189, in absolute terms correlation has little effect on the UMVCUE. However, note the piecewise nature of the UMVCUE: it increases for $-1<\rho <-0.58$, decreases for $-0.57<\rho <-0.33$, remains constant for $-0.33<\rho <0.1,$ and then (rapidly) decreases again for $\rho >0.1$. Also note that this time, there *is* a large discrepancy between the stage 1 and stage 2 log ORs (1.51 and 1.38, respectively). The fact that the UMVCUE hardly varies indicates that the substantial correction to the stage 1 estimate is in fact robust to correlation.

As for the second plot for SNP rs4958847, again the correlation hardly changes the value of the UMVCUE (in fact, the UMVCUE remains constant until $\rho > 0.5$). However, this should be expected since the stage 1 and stage 2 estimates for the OR were very similar (1.35 and 1.36, respectively).

*Constructing CIs*. After constructing a point estimate for the OR, it is natural to construct CIs as well. We use a parametric bootstrap procedure inspired by Bowden and Glimm (2008) and Pepe *and others* (2009), where we assume that the true log OR mean $\mu _j = \hat {U}_j$ (the UMVCUE calculated from the observed data).

Starting with the SNPs on chromosomal region 5q33, these had the fifth and seventh stage 1 ranks. Given the observed stage 1 log ORs ${\boldsymbol x} = (x_1, \ldots , x_{11})$ and the assumed correlation coefficient $\rho $ between $X_5$ and $X_7$, we use the following procedure:

1. Simulate “bootstrapped” stage 1 data $X_5^{(B)}, X_7^{(B)}$ by drawing from a bivariate normal distribution
  \[ \begin{pmatrix} X_5^{(B)} \\
X_7^{(B)} \end{pmatrix} \sim N \left( \begin{pmatrix} \hat{U}_5 \\
\hat{U}_7 \end{pmatrix} , \begin{pmatrix} \sigma_5^2 & \rho \sigma_5 \sigma_7\\
\rho \sigma_5 \sigma_7 & \sigma_7^{2} \end{pmatrix}\right), \]
  conditional on $|x_4|/\sigma _4 \geq |X_5^{(B)}|/\sigma _5 \geq |x_6|/\sigma _6$ and $|x_6|/\sigma _6 \geq |X_7^{(B)}|/\sigma _7 \geq |x_8|/\sigma _8$.
2. Simulate bootstrapped stage 2 data $Y_5^{(B)}, Y_7^{(B)}$ from independent normal distributions $Y_5^{(B)} \sim N( \hat {U}_5, \sigma _5^2)$ and $Y_7^{(B)} \sim N( \hat {U}_7, \sigma _7^2)$.
3. Calculate the bootstrapped UMVCUEs $\hat {U}_5^{(B)}, \hat {U}_7^{(B)}$ using $X_5^{(B)}, X_7^{(B)}, Y_5^{(B)}, Y_7^{(B)}$ and the original observed values $x_1, \ldots , x_4, x_6, x_8, \ldots , x_{11}$.

These steps are then repeated for a large value of $B$, with the $\alpha /2$ and $(1-\alpha /2)$ empirical quantiles then used as the $(1-\alpha )\%$ CIs for $\hat {U}_5$ and $\hat {U}_7$. Bootstrapped CIs for the MLE, as well as the Zhong & Prentice estimator (ZP) are also then straightforwardly available.

Further details of how the variables are generated in step 1 can be found in Section 7 of the supplementary materials (available at *Biostatistics* online), as well as an extension to the general multivariate case.

As for the SNPs on chromosomal region 5p13, these had the first and second stage 1 ranks. The same bootstrap procedure described above can be used, except that in step 1, we condition on $|X_1^{(B)}|/\sigma _1 \geq |X_2^{(B)}|/\sigma _2 \geq |x_3|/\sigma _3$.

**Fig. 4.**
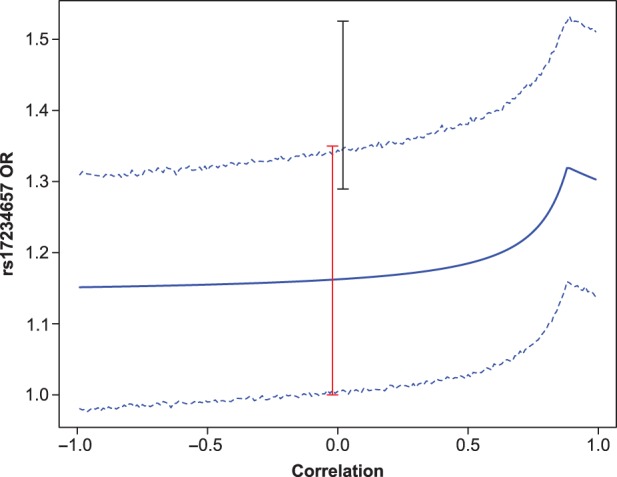
The UMVCUE for the OR of the highest ranked SNP (rs17234657) as a function of the correlation coefficient with the second-ranked SNP (rs9292777). Also plotted are the 95% bootstrapped CIs for the UMVCUE, using 10 000 bootstrapped replicates per data point. The 95% CIs for the stage 2 OR (in red) and the MLE (in black) are displayed for comparison purposes.

Figure 4 shows the bootstrapped 95% CIs for SNP rs17234657 on chromosomal region 5p13. When the correlation $\rho = 0$, the CI for the UMVCUE is virtually identical to that achieved by just using the stage 2 data. However, we see that, for $\rho >0.5,$ the CIs become appreciably different, since the stage 2 OR ignores the correlation.

Table 2 compares the bootstrapped 95% CIs for the various estimators for the two highest ranked SNPs (rs17234657 and rs9292777) when the correlation $\rho = 0$. For the SNP rs9292777, there is a 12% reduction in the CI width (from 0.304 to 0.268) when using the UMVCUE compared to just using the stage 2 data. For both SNPs, the ZP estimator and the MLE give very similar CIs.

**Table 2.** Bootstrapped $95\%$ CIs for the ORs of the two highest ranked SNPs when the correlation $\rho = 0$. There were $10\,000$ bootstrapped replicates used

	rs17234657	rs9292777
Stage 2	(1.002, 1.348)	(1.243, 1.547)
UMVCUE	(1.003, 1.348)	(1.299, 1.566)
ZP	(1.289, 1.529)	(1.308, 1.487)
MLE	(1.290, 1.526)	(1.307, 1.485)

## 5. Discussion

In this paper, we present a general framework for accounting for selection and correlation in two-stage trials, with an emphasis on the GWAS setting. We achieve this by deriving the UMVCUE in the multivariate normal setting with an arbitrary known covariance structure. Our framework relaxes the assumption present in the literature that the stage 1 population parameter estimates are independent.

As the bivariate normal simulation study demonstrated, it is important to correctly account for correlation. Indeed, if the correlation coefficient is not close to zero, then the UMVCUE that ignores correlation can be substantially biased. Due to the bias–variance trade-off, the MSE of the UMVCUE is greater than that of the MLE, but is substantially less than the MSE of using the (unbiased) stage 2 data alone.

The GWAS example showed how our estimation strategy can be applied in practice. We also described how to construct CIs using a parametric bootstrap procedure. Our results demonstrate how correcting for correlation is necessary in the GWAS setting. Indeed, for the Parkes *and others* (2007) data, the OR estimate for the highest ranking SNP varied substantially for high positive values of the correlation coefficient.

In the GWAS setting, our framework assumes that the LD structure of the SNPs is known. In practice, we would envisage using large external datasets such as the International HapMap Project (The International HapMap Consortium, 2003) to give an estimate of SNP LD. Alternatively, the correlation could be estimated based on the stage 1 data itself if individual participant data were available, but further research is needed to give guidance as to whether doing so would induce bias into our UMVCUE and/or appreciably increase the MSE.

Even if it is not possible to have reliable estimates of the correlation between the (log) ORs, a sensitivity analysis (like the one we carried out) is still useful to probe the robustness of the reported ORs to any LD between the SNPs.

Due to the structure of the Parkes *and others* (2007) data, our results in Section 4.2 focused on the bivariate setting. Of course, in other GWAS studies the data may mean that it is necessary to consider multiple (i.e. $K > 2$) SNPs in a local region that are in LD. Our UMVCUE, although still tractable, would then have a much more complex dependence on $K(K-1)/2$ correlation coefficients.

We note that some progress was made to account for correlated outcomes by Sill and Sampson (2007), who looked at two-stage trials with mid-trial treatment selection based on a surrogate endpoint. They derived the UMVCUE for the final endpoint when the surrogate and final endpoint follow a bivariate normal distribution. However, our framework is a more general one, and allows application to other trial settings. Indeed, as current work, we have applied our estimator to two-stage adaptive seamless phase II/III clinical trials, allowing for unequal stage 1 (and stage 2) variances as well as generalized selection rules.

One of the limitations of our work is the construction of CIs. In the bivariate case, we can simply use a parametric bootstrap procedure. As for the general multivariate case, in the supplementary materials (available at *Biostatistics* online) we show theoretically how to extend our method. However, it is not clear what the coverage of such CIs would be in practice. An alternative approach could be based on the work of Sampson and Sill (2005), who developed exact CIs in the independent normal setting. It may be possible to extend their analysis to correlated multivariate normal outcomes.

In the framework for our UMVCUE, we treat the stage 2 data as coming from independent univariate normal distributions, i.e. we ignore the potential stage 2 correlation structure. As current work, we are deriving efficient unbiased estimators that make use of, and fully account for, all the available correlated stage 2 outcomes. However, preliminary results appear to show that no UMVCUE exists for this case, despite the obvious appeal of making use of more data, when available.

Finally, another extension would be trials with more than two stages. Bowden and Glimm (2014) derived Rao–Blackwellized estimators for multistage drop-the-loser trials assuming independent normal outcomes, and this could be a starting point for looking at multistage trials with multiply correlated outcomes.

## Supplementary material

Supplementary Material is available at http://biostatistics.oxfordjournals.org.

## Funding

This work was supported by a Medical Research Council Methodology Research Fellowship (grant number MR/N501906/1 to J.B.). Funding to pay the Open Access publication charges for this article was provided by the MRC BSU, grant codes MC_UP_1302/2 and MC_UP_1302/3.

## Supplementary Material

Supplementary Data
