# Supplementary material for: Accounting for selection and correlation in the analysis of two-stage genome-wide association studies
Source: Biostatistics. 2016 Mar 18;17(4):634–49. doi: 10.1093/biostatistics/kxw012 (PMC5031943; doi:10.1093/biostatistics/kxw012)
Supplement: Supplementary Data [file supp_17_4_634__index.html]

Accounting for selection and correlation in the analysis of two-stage genome-wide association studies — Supplementary Data 

# Accounting for selection and correlation in the analysis of two-stage genome-wide association studies

## Supplementary Data

Supplementary Data

**Files in this Supplementary Material:**

- Supplementary Data
