# Supplementary material for: Accounting for selection and correlation in the analysis of two-stage genome-wide association studies
Source: Biostatistics. 2016 Mar 18;17(4):634–49. doi: 10.1093/biostatistics/kxw012 (PMC5031943; doi:10.1093/biostatistics/kxw012)
Supplement: Supplementary Data [file supp_kxw012_kxw012supp.pdf]

Biostatistics (2016), *pp.* 1–14
doi:10.1093/biostatistics/kxw012

Accounting for selection and correlation in the analysis of two-stage genome-wide association studies: supplementary materials

DAVID S. ROBERTSON*
MRC Biostatistics Unit, IPH Forvie Site, Robinson Way, Cambridge CB2 0SR
david.robertson@mrc-bsu.cam.ac.uk

A. TOBY PREVOST
Imperial College London, 1st Floor, Stadium House, 68 Wood Lane, London W12 7RH

JACK BOWDEN
MRC Integrative Epidemiology Unit, University of Bristol, Oakfield House, Bristol BS8 2BN
MRC Biostatistics Unit, IPH Forvie Site, Robinson Way, Cambridge CB2 0SR

1. Proof of the completeness and sufficiency of Z

Theorem 1.1 The statistic $\boldsymbol{Z} = (Z_1, \ldots, Z_K)$ is sufficient and complete for $\boldsymbol{\mu} = (\mu_1, \ldots, \mu_K)$, where

$$Z_i = X_i + \frac{V_{1i}}{\tau^2} Y \tag{1.1}$$

for $i = 1, \ldots, K$.

Proof.

The joint distribution of $(\boldsymbol{X}, Y)$ given Q has the density

$$f_Q(\boldsymbol{x}, y) = p_Q^{-1} I_Q(\boldsymbol{x}) g(\boldsymbol{x}) \frac{1}{\tau} \phi\left(\frac{y - \mu_1}{\tau}\right) \tag{1.2}$$

where p_Q is the probability of the event Q occurring, $I_Q(\boldsymbol{x})$ is the indicator function for Q, ϕ is the pdf of the standard (univariate) normal distribution and

$$g(\boldsymbol{x}) = \frac{1}{\sqrt{(2\pi)^K |V|}} \exp\left[-\frac{1}{2}(\boldsymbol{x} - \boldsymbol{\mu})^T P (\boldsymbol{x} - \boldsymbol{\mu})\right]$$

is the (non-degenerate) multivariate normal pdf.

*To whom correspondence should be addressed.

© The Author 2016. Published by Oxford University Press. All rights reserved. For permissions, please e-mail: journals.permissions@oup.com

Now the exponent in equation (1.2) is:

$$\frac{1}{2}\left[\boldsymbol{x}^T P\boldsymbol{\mu} + \boldsymbol{\mu}^T P\boldsymbol{x} - \boldsymbol{\mu}^T P\boldsymbol{\mu} - \frac{1}{\tau^2}\mu_1^2 + \frac{2}{\tau^2}y\mu_1 - \boldsymbol{x}^T P\boldsymbol{x} - \frac{1}{\tau^2}y^2\right]$$
$$= \frac{1}{2}\left[2\left(\sum_{i=1}^K P_{1i}x_i + \frac{1}{\tau^2}y\right)\mu_1 + 2\sum_{i=2}^K\left(\sum_{j=1}^K P_{ij}x_j\right)\mu_i - \boldsymbol{\mu}^T P\boldsymbol{\mu} - \frac{1}{\tau^2}\mu_1^2 + K(\boldsymbol{x}, y)\right]$$

where $K(\boldsymbol{x}, y) = -\boldsymbol{x}^T P\boldsymbol{x} - \frac{1}{\tau^2}y^2$ is independent of $\boldsymbol{\mu}$.

Hence by the factorisation criteria, the statistic $\boldsymbol{T} = (T_1, \ldots, T_K)$ is sufficient for $\boldsymbol{\mu}$, where

$$T_1 = \sum_{i=1}^K P_{1i}x_i + \frac{1}{\tau^2}y$$
$$T_i = \sum_{j=1}^K P_{ij}x_j \qquad \text{for } i = 2, \ldots, K.$$

Since we are working with an exponential family (and the natural parameter space contains a K-dimensional rectangle) then in fact $\boldsymbol{T}$ is also a complete statistic – see for example Theorem 4.3.1 in Lehmann and Romano (2005).

Now we form linear combinations of $\boldsymbol{T}$ in order to get the required sufficient statistic. Firstly, if $V_{1i} \neq 0$, for $i \in 1, \ldots, K$, then consider the following:

$$\tilde{Z}_i = T_1 + \sum_{j=2}^K \frac{V_{ij}}{V_{1i}}T_j$$
$$= \sum_{j=1}^K P_{1j}x_j + \frac{1}{\tau^2}y + \frac{1}{V_{1i}}\sum_{j=2}^K V_{ij}\sum_{k=1}^K P_{jk}x_k$$
$$= \sum_{j=1}^K P_{1j}x_j + \frac{1}{\tau^2}y + \frac{1}{V_{1i}}\left[\sum_{j,k=1}^K V_{ij}P_{jk}x_k - V_{1i}\sum_{k=1}^K P_{1k}x_k\right]$$
$$= \frac{1}{\tau^2}y + \frac{x_i}{V_{1i}} = \frac{1}{V_{1i}}\left[x_i + \frac{V_{1i}}{\tau^2}y\right].$$

Hence $Z_i = X_i + \frac{V_{1i}}{\tau^2}Y$ is a sufficient statistic for μ_i. Note that in particular, we must have $Z_1 = X_1 + \frac{V_{11}}{\tau^2} = X_1 + \frac{\sigma_1^2}{\tau^2}Y$, since $V_{11} = \sigma_1^2 > 0$.

If however $V_{1i} = 0$, then consider

$$\tilde{Z}_i = \sum_{j=2}^K V_{ij}T_j = \sum_{j=2}^K V_{ij}\sum_{k=1}^K P_{jk}x_k$$
$$= \sum_{j,k=1}^K V_{ij}P_{jk}x_k = x_i.$$

Hence $Z_i = X_i + \frac{V_{1i}}{\tau^2}Y$ is a sufficient statistic for μ_i when $V_{1i} = 0$.

Since $\boldsymbol{Z}$ is just a linear transformation of $\boldsymbol{T}$, then it too is sufficient and complete, as claimed.

□

2. Derivation of the UMVCUE in the multivariate normal setting

Theorem 2.1 The UMVCUE for μ_1 given $Q = \{\boldsymbol{X} : X_1 > X_2 > \cdots > X_K\}$ is

$$\hat{U} = \frac{\tau^2 Z_1}{\sigma_1^2 + \tau^2} - \frac{\tau^2}{\sqrt{\sigma_1^2 + \tau^2}} \frac{\phi(W_1) - \phi(W_2)}{\Phi(W_1) - \Phi(W_2)} \tag{2.3}$$

where

$$\begin{aligned} W_i &= \frac{k_i\sqrt{\sigma_1^2 + \tau^2}}{\tau^2} - \frac{Z_1}{\sqrt{\sigma_1^2 + \tau^2}} \qquad \text{for } i = 1, 2 \\ k_1 &= \min(A_1), \quad k_2 = \max(A_2), \\ A_1 &= \left\{ \frac{\tau^2(Z_j - Z_{j+1})}{V_{1j} - V_{1,j+1}} : V_{1j} > V_{1,j+1} \,;\, j = 1, \ldots, K-1 \right\}, \\ A_2 &= \left\{ \frac{\tau^2(Z_j - Z_{j+1})}{V_{1j} - V_{1,j+1}} : V_{1j} < V_{1,j+1} \,;\, j = 1, \ldots, K-1 \right\} \end{aligned}$$

and we define $\min\{\emptyset\} = +\infty$ and $\max\{\emptyset\} = -\infty$.

Proof.
As before, the joint distribution of $(\boldsymbol{X}, Y)$ given Q has the density

$$f_Q(\boldsymbol{x}, y) = p_Q^{-1} I_Q(\boldsymbol{x}) g(\boldsymbol{x}) \frac{1}{\tau}\phi\left(\frac{y - \mu_1}{\tau}\right).$$

Now from the definition of $\boldsymbol{Z}$, we have the following relations:

$$X_i = Z_i - \frac{V_{1i}}{\tau^2} Y \qquad \text{for } i = 1, \ldots, K \tag{2.4}$$

which can be written as $\boldsymbol{X} = \boldsymbol{Z} - \frac{Y}{\tau^2}\boldsymbol{V_1}$.

Hence the joint distribution of $(Y, \boldsymbol{Z})$ and Y given Q has the density

$$f_Q(y, \boldsymbol{z}) = p_Q^{-1} I_Q(\boldsymbol{z}) g\left(\boldsymbol{z} - \frac{y}{\tau^2}\boldsymbol{V_1}\right) \frac{1}{\tau}\phi\left(\frac{y - \mu_1}{\tau}\right).$$

Integrating over the support of Y, denoted A, gives the pdf for $\boldsymbol{Z}$ given Q as

$$f_Q(\boldsymbol{z}) = p_Q^{-1} I_Q(\boldsymbol{z}) \int_{y \in A} g\left(\boldsymbol{z} - \frac{y}{\tau^2}\boldsymbol{V_1}\right) \frac{1}{\tau}\phi\left(\frac{y - \mu_1}{\tau}\right) dy$$

Hence the conditional distribution of $Y|\boldsymbol{Z}, Q$ has density

$$\begin{aligned} f_Q(y|\boldsymbol{z}) &= \frac{f_Q(y, \boldsymbol{z})}{f_Q(\boldsymbol{z})} \\ &= \frac{g\left(\boldsymbol{z} - \frac{y}{\tau^2}\boldsymbol{V_1}\right) \frac{1}{\tau}\phi\left(\frac{y-\mu_1}{\tau}\right)}{\int_{y \in A} g\left(\boldsymbol{z} - \frac{y}{\tau^2}\boldsymbol{V_1}\right) \frac{1}{\tau}\phi\left(\frac{y - \mu_1}{\tau}\right) dy} \end{aligned}$$

Since Y is an unbiased estimator for μ_1, and $\boldsymbol{Z}$ is a complete sufficient statistic, then the UMVCUE is given by $\hat{U} = E\left[Y|\boldsymbol{Z}, Q\right]$. Therefore, we can calculate $\hat{U}$ directly as

$$\hat{U} = \frac{\displaystyle\int_{y\in A} y\, g\left(\boldsymbol{z} - \frac{y}{\tau^2}\boldsymbol{V_1}\right)\frac{1}{\tau}\phi\left(\frac{y-\mu_1}{\tau}\right)dy}{\displaystyle\int_{y\in A} g\left(\boldsymbol{z} - \frac{y}{\tau^2}\boldsymbol{V_1}\right)\frac{1}{\tau}\phi\left(\frac{y-\mu_1}{\tau}\right)dy}$$

In order to simplify this expression for $\hat{U}$, we first find the support of Y. Conditioning on the event Q means that $X_i > X_{i+1}$ for $i = 1, \ldots, K-1$. From equations (2.4) this gives

$$\begin{aligned} X_i > X_{i+1} &\implies Z_i - \frac{V_{1i}}{\tau^2}Y > Z_{i+1} - \frac{V_{1,i+1}}{\tau^2}Y \\ &\implies (V_{1i} - V_{1,i+1})\,Y < \tau^2\left(Z_i - Z_{i+1}\right). \end{aligned} \tag{2.5}$$

If $V_{1i} > V_{1,i+1}$ then equation (2.5) implies that $Y < \frac{\tau^2(Z_i - Z_{i+1})}{V_{1i}-V_{1,i+1}}$. Conversely, if $V_{1i} < V_{1,i+1}$ then equation (2.5) implies that $Y > \frac{\tau^2(Z_i - Z_{i+1})}{V_{1i}-V_{1,i+1}}$. However, when $V_{1i} = V_{1,i+1}$, then there is no restriction contributed to the support of Y.

Putting all of the above inequalities together, we find that $k_2 < Y < k_1$, where

$$\begin{aligned} &k_1 = \min(A_1),\ k_2 = \max(A_2) \\ &A_1 = \left\{\frac{\tau^2(Z_j - Z_{j+1})}{V_{1j} - V_{1,j+1}} : V_{1j} > V_{1,j+1}\,;\ j = 1, \ldots, K-1\right\} \\ &A_2 = \left\{\frac{\tau^2(Z_j - Z_{j+1})}{V_{1j} - V_{1,j+1}} : V_{1j} < V_{1,j+1}\,;\ j = 1, \ldots, K-1\right\} \end{aligned}$$

and we define $\min\{\emptyset\} = +\infty$ and $\max\{\emptyset\} = -\infty$.

Note that we always have $k_1 < k_2$. Indeed, suppose

$$\frac{\tau^2(Z_j - Z_{j+1})}{V_{1j} - V_{1,j+1}} < \frac{\tau^2(Z_k - Z_{k+1})}{V_{1k} - V_{1,k+1}}$$

where $V_{1j} > V_{1,j+1}$ and $V_{1k} < V_{1,k+1}$. This implies that

$$\begin{aligned} &(Z_j - Z_{j+1})(V_{1k} - V_{1,k+1}) > (Z_k - Z_{k+1})(V_{1j} - V_{1,j+1}) \\ \implies &\left[X_j - X_{j+1} + \frac{1}{\tau^2}(V_{1j} - V_{1,j+1})Y\right](V_{1k} - V_{1,k+1}) > \\ &\qquad\left[X_k - X_{k+1} + \frac{1}{\tau^2}(V_{1k} - V_{1,k+1})Y\right](V_{1j} - V_{1,j+1}) \\ \implies &(X_j - X_{j+1})(V_{1k} - V_{1,k+1}) > (X_k - X_{k+1})(V_{1j} - V_{1,j+1}) \end{aligned}$$

which is a contradiction since the LHS is less than zero, whereas the RHS is greater than zero.

Hence, we have the following expression for $\hat{U}$:

$$\hat{U} = \frac{\displaystyle\int_{k_2}^{k_1} y\, g\left(\boldsymbol{z} - \frac{y}{\tau^2}\boldsymbol{V_1}\right)\frac{1}{\tau}\phi\left(\frac{y-\mu_1}{\tau}\right)dy}{\displaystyle\int_{k_2}^{k_1} g\left(\boldsymbol{z} - \frac{y}{\tau^2}\boldsymbol{V_1}\right)\frac{1}{\tau}\phi\left(\frac{y-\mu_1}{\tau}\right)dy} \tag{2.6}$$

Now consider the exponent of $g\left(\boldsymbol{z} - \frac{y}{\tau^2}\boldsymbol{V_1}\right)\phi\left(\frac{y-\mu_1}{\tau}\right)$. This will be equal to

$$
\begin{aligned}
&-\frac{1}{2}\left[\left(\boldsymbol{z} - \frac{y}{\tau^2}\boldsymbol{V_1} - \boldsymbol{\mu}\right)^T P\left(\boldsymbol{z} - \frac{y}{\tau^2}\boldsymbol{V_1} - \boldsymbol{\mu}\right) + \frac{1}{\tau^2}(y-\mu_1)^2\right] \\
&= \frac{1}{2}\left[\left(\boldsymbol{z} - \frac{y}{\tau^2}\boldsymbol{V_1}\right)^T P\boldsymbol{\mu} + \boldsymbol{\mu}^T P\left(\boldsymbol{z} - \frac{y}{\tau^2}\boldsymbol{V_1}\right) - \boldsymbol{\mu}^T P\boldsymbol{\mu} - \frac{1}{\tau^2}y^2 + \frac{2}{\tau^2}y\mu_1 - \frac{1}{\tau^2}\mu_1^2\right. \\
&\qquad\left. - \left(\boldsymbol{z} - \frac{y}{\tau^2}\boldsymbol{V_1}\right)^T P\left(\boldsymbol{z} - \frac{y}{\tau^2}\boldsymbol{V_1}\right)\right] \\
&= \frac{1}{2}\left[2\left\{\sum_{i=1}^{K} P_{1i}\left(z_i - \frac{V_{1i}}{\tau^2}y\right) + \frac{1}{\tau^2}y\right\}\mu_1 + 2\sum_{i=2}^{K}\left\{\sum_{j=1}^{K} P_{ij}\left(z_j - \frac{V_{1j}}{\tau^2}y\right)\right\}\mu_i\right. \\
&\qquad\left. -\frac{1}{\tau^2}y^2 - \sum_{i,j=1}^{K}\frac{y^2}{\tau^4}V_{1i}P_{ij}V_{1j} + \frac{2}{\tau^2}\left\{\sum_{i,j=1}^{K} z_i P_{ij} V_{1j} y\right\} + K_1(\boldsymbol{z})\right] \\
&= \frac{1}{2}\left[2\left(\sum_{i=1}^{K} P_{1i}z_i\right)\mu_1 + 2\sum_{i=2}^{K}\left(\sum_{j=1}^{K} P_{ij}z_j\right)\mu_i - \frac{1}{\tau^2}y^2 - \frac{y^2}{\tau^4}\sigma_1^2 + \frac{2}{\tau^2}z_1 y + K_1(\boldsymbol{z})\right] \\
&= -\frac{1}{2}\left[\frac{1}{\tau^2}\left(1 + \frac{\sigma_1^2}{\tau^2}\right)y^2 - \frac{2}{\tau^2}z_1 y + K_2(\boldsymbol{z})\right] \\
&= -\frac{1}{2\eta^2}\left(y - \frac{\tau^2 z_1}{\sigma_1^2 + \tau^2}\right)^2 + K_3(\boldsymbol{z})
\end{aligned}
$$

where the $K_i(\boldsymbol{z})$ are independent of Y for $i = 1, 2, 3$ and $\eta = \frac{\tau^2}{\sqrt{\sigma_1^2+\tau^2}}$.

Noting that terms of $\exp[K_3(\boldsymbol{z})]$ will cancel out in the numerator and denominator of equation (2.6), we have

$$
\hat{U} = \frac{\displaystyle\int_{k_2}^{k_1} y\,\frac{1}{\sqrt{2\pi\eta^2}}\exp\left[-\frac{1}{2\eta^2}\left(y - \frac{\tau^2 z_1}{\sigma_1^2+\tau^2}\right)^2\right]dy}{\displaystyle\int_{k_2}^{k_1}\frac{1}{\sqrt{2\pi\eta^2}}\exp\left[-\frac{1}{2\eta^2}\left(y - \frac{\tau^2 z_1}{\sigma_1^2+\tau^2}\right)^2\right]dy} \tag{2.7}
$$

where we have multiplied both the numerator and denominator by $\frac{1}{\sqrt{2\pi\eta^2}}$.

Now the function $\frac{1}{\sqrt{2\pi\eta^2}}\exp\left[-\frac{1}{2\eta^2}\left(y - \frac{\tau^2}{\sigma_1^2+\tau^2}z_1\right)^2\right]$ corresponds to the pdf of a $N\left(\frac{\tau^2}{\sigma_1^2+\tau^2}z_1, \eta^2\right)$ random variable. Hence the denominator in equation (2.7) is equal to

$$
\Phi\left(\frac{k_1 - \frac{\tau^2}{\sigma_1^2+\tau^2}z_1}{\eta}\right) - \Phi\left(\frac{k_2 - \frac{\tau^2}{\sigma_1^2+\tau^2}z_1}{\eta}\right).
$$

As for the numerator in equation (2.7), this integral can be solved by using the appropriate

substitution $v = -\frac{1}{2\eta^2}\left(y - \frac{\tau^2}{\sigma_1^2+\tau^2}z_1\right)^2$ to give

$$-\eta\left[\phi\left(\frac{k_1 - \frac{\tau^2}{\sigma_1^2+\tau^2}z_1}{\eta}\right) - \phi\left(\frac{k_2 - \frac{\tau^2}{\sigma_1^2+\tau^2}z_1}{\eta}\right)\right]$$
$$+\frac{\tau^2 z_1}{\sigma_1^2+\tau^2}\left[\Phi\left(\frac{k_1 - \frac{\tau^2}{\sigma_1^2+\tau^2}z_1}{\eta}\right) - \Phi\left(\frac{k_2 - \frac{\tau^2}{\sigma_1^2+\tau^2}z_1}{\eta}\right)\right].$$

Putting everything together gives the expression in equation (2.3) and completes the proof.

□

3. Comparison with the estimator of Bowden and Glimm (2008)

If we set the off-diagonal terms of the covariance matrix equal to 0, the sufficient, complete statistic Z_{ij} becomes

$$Z_{ij} = \begin{cases} X_j + \frac{\sigma_j^2}{\tau_j^2}Y_j & i = j \\ X_i & i \neq j \end{cases}$$

Bowden and Glimm (2008), derived the following estimator for μ_j conditional on Q:

$$\tilde{\mu}_j = \frac{\tau_j^2 X_j + \sigma_j^2 Y_j}{\sigma_j^2 + \tau_j^2} - \frac{\tau_j^2}{\sqrt{\sigma_j^2+\tau_j^2}}\frac{\phi(W_{j,j+1}) - \phi(W_{j,j-1})}{\Phi(W_{j,j+1}) - \Phi(W_{j,j-1})}$$

where

$$W_{j,s} = \frac{\sqrt{\sigma_j^2+\tau_j^2}}{\sigma_j^2}\left(\frac{\tau_j^2 X_j + \sigma_j^2 Y_j}{\sigma_j^2+\tau_j^2} - X_s\right).$$

Note that by definition, $W_{1,0} = -\infty$, and $W_{K,K+1} = \infty$.

As for our estimator, firstly note that $V_{ij} = 0$ for $i \neq j$, meaning that $V_{ij} = V_{i+1,j} = 0$ for $i \notin \{j, j-1\}$.

Considering the case when $1 < j < K$, then the sets A_1 and A_2 only have the terms with $i = j$ and $i = j-1$ respectively. This implies that

$$k_1 = \frac{\tau_j^2\,(Z_{jj} - X_{j+1})}{\sigma_j^2},\ k_2 = \frac{\tau_j^2\,(Z_{jj} - X_{j-1})}{\sigma_j^2}$$

which gives

$$\begin{aligned} W_1 &= \frac{k_1\sqrt{\sigma_j^2+\tau_j^2}}{\tau_j^2} - \frac{Z_{jj}}{\sqrt{\sigma_j^2+\tau_j^2}} \\ &= \frac{\sqrt{\sigma_j^2+\tau_j^2}}{\sigma_j^2}\left(Z_{jj} - X_{j+1} - \frac{\sigma_j^2 Z_{jj}}{\sigma_j^2+\tau_j^2}\right) \\ &= \frac{\sqrt{\sigma_j^2+\tau_j^2}}{\sigma_j^2}\left(\frac{\tau_j^2 X_j + \sigma_j^2 Y_j}{\sigma_j^2+\tau_j^2} - X_{j+1}\right) \end{aligned}$$

and similarly

$$W_2 = \frac{\sqrt{\sigma_j^2 + \tau_j^2}}{\sigma_j^2} \left(\frac{\tau_j^2 X_j + \sigma_j^2 Y_j}{\sigma_j^2 + \tau_j^2} - X_{j-1} \right).$$

If $j = 1$, then the set A_2 is empty, so $k_2 = -\infty$ and hence $W_2 = -\infty$. This agrees with the definition of $W_{1,0} = -\infty$. Finally, if $j = K$ then the set A_1 is empty, so $k_1 = \infty$ and hence $W_1 = \infty$.

Hence our estimator

$$\hat{U}_j = \frac{\tau_j^2 Z_{jj}}{\sigma_j^2 + \tau_j^2} - \frac{\tau_j^2}{\sqrt{\sigma_j^2 + \tau_j^2}} \frac{\phi(W_1) - \phi(W_2)}{\Phi(W_1) - \Phi(W_2)}$$

is the same as Bowden and Glimm's for all $j \in \{1, \ldots, K\}$.

4. Derivation of the UMVCUE for the one-sided test

Theorem 4.1 The UMVCUE for μ_j given

$$Q_1 = \left\{ \boldsymbol{X} : \frac{X_1}{\sigma_1} \geqslant \frac{X_2}{\sigma_2} \geqslant \cdots \geqslant \frac{X_K}{\sigma_K} \geqslant \Phi^{-1}(1 - p_{\text{crit}}) \right\}.$$

is

$$\hat{U}_j = \frac{\tau_j^2 Z_{jj}}{\sigma_j^2 + \tau_j^2} - \frac{\tau_j^2}{\sqrt{\sigma_j^2 + \tau_j^2}} \frac{\phi(W_1) - \phi(W_2)}{\Phi(W_1) - \Phi(W_2)} \tag{4.8}$$

where

$$W_i = \frac{k_i \sqrt{\sigma_j^2 + \tau_j^2}}{\tau_j^2} - \frac{Z_{jj}}{\sqrt{\sigma_j^2 + \tau_j^2}} \qquad \text{for } i = 1, 2$$

$$k_1 = \min(A_1, A_2), \quad k_2 = \max(A_3, A_4),$$

$$A_1 = \left\{ \frac{\tau_j^2(\sigma_{i+1} Z_{ij} - \sigma_i Z_{i+1,j})}{\sigma_{i+1} V_{ij} - \sigma_i V_{i+1,j}} : \sigma_{i+1} V_{ij} > \sigma_i V_{i+1,j} \; ; \; i = 1, \ldots, K-1 \right\},$$

$$A_2 = \left\{ \frac{\tau_j^2}{V_{Kj}} \left[Z_{Kj} - \sigma_K \Phi^{-1}(1 - p_{\text{crit}}) \right] : V_{Kj} > 0 \right\},$$

$$A_3 = \left\{ \frac{\tau_j^2(\sigma_{i+1} Z_{ij} - \sigma_i Z_{i+1,j})}{\sigma_{i+1} V_{ij} - \sigma_i V_{i+1,j}} : \sigma_{i+1} V_{ij} < \sigma_i V_{i+1,j} \; ; \; i = 1, \ldots, K-1 \right\},$$

$$A_4 = \left\{ \frac{\tau_j^2}{V_{Kj}} \left[Z_{Kj} - \sigma_K \Phi^{-1}(1 - p_{\text{crit}}) \right] : V_{Kj} < 0 \right\}$$

and we define $\min\{\emptyset\} = +\infty$ and $\max\{\emptyset\} = -\infty$.

Proof.

Everything follows through as in Theorem 2.1, except that the support of Y changes and hence (k_1, k_2) changes too.

Conditioning on the event Q_1 means that $\frac{X_i}{\sigma_i} \geqslant \frac{X_{i+1}}{\sigma_{i+1}}$ for $i = 1, \dots, K-1$. Using equations (1.1), this gives the following

$$\begin{aligned} \frac{X_i}{\sigma_i} \geqslant \frac{X_{i+1}}{\sigma_{i+1}} &\implies \sigma_{i+1}\left(Z_{ij} - \frac{V_{ij}}{\tau_j^2}Y_j\right) \geqslant \sigma_i \left(Z_{i+1,j} - \frac{V_{i+1,j}}{\tau_j^2}Y_j\right) \\ &\implies (\sigma_i V_{i+1,j} - \sigma_{i+1}V_{ij})Y_j \leqslant \tau_j^2(\sigma_{i+1}Z_{ij} - \sigma_i Z_{i+1,j}). \end{aligned}$$

Hence if $\sigma_{i+1}V_{ij} > \sigma_i V_{i+1,j}$ then $Y \leqslant \frac{\tau_j^2(\sigma_{i+1}Z_{ij} - \sigma_i Z_{i+1,j})}{\sigma_{i+1}V_{ij} - \sigma_i V_{i+1,j}}$ with the inequality sign reversed if $\sigma_{i+1}V_{ij} < \sigma_i V_{i+1,j}$. If $\sigma_{i+1}V_{ij} = \sigma_i V_{i+1,j}$ there is no restriction contributed to the support of Y.

Finally, for $i = K$ we have

$$\begin{aligned} \frac{X_K}{\sigma_K} \geqslant \Phi^{-1}(1 - p_{\text{crit}}) &\implies Z_{Kj} - \frac{V_{Kj}}{\tau_j^2}Y_j > \sigma_K \Phi^{-1}(1 - p_{\text{crit}}) \\ &\implies V_{Kj}Y_j \leqslant \tau_j^2\left[Z_{Kj} - \sigma_K \Phi^{-1}(1 - p_{\text{crit}})\right]. \end{aligned}$$

□

5. Derivation of the UMVCUE for the two-sided test

Theorem 5.1 The UMVCUE for μ_j given

$$Q_2 = \left\{\boldsymbol{X} : \frac{|X_1|}{\sigma_1} \geqslant \frac{|X_2|}{\sigma_2} \geqslant \cdots \geqslant \frac{|X_K|}{\sigma_K} \geqslant \Phi^{-1}(1 - p_{\text{crit}}/2)\right\}$$

is

$$\hat{U}_j = \frac{\tau_j^2 Z_{jj}}{\sigma_j^2 + \tau_j^2} - \frac{\tau_j^2}{\sqrt{\sigma_j^2 + \tau_j^2}} \frac{\sum_{i=1}^M \phi(W_{1i}) - \phi(W_{2i})}{\sum_{i=1}^M \Phi(W_{1i}) - \Phi(W_{2i})} \tag{5.9}$$

where

$$\begin{aligned} W_{1i} &= \frac{b_i\sqrt{\sigma_j^2 + \tau_j^2}}{\tau_j^2} - \frac{Z_{jj}}{\sqrt{\sigma_j^2 + \tau_j^2}}, \qquad W_{2i} = \frac{a_i\sqrt{\sigma_j^2 + \tau_j^2}}{\tau_j^2} - \frac{Z_{jj}}{\sqrt{\sigma_j^2 + \tau_j^2}}, \\ \bigcup_{i=1}^M [a_i, b_i] &= \left(\bigcap_{i=1}^{K-1} (A_{1i} \cap A_{2i}) \cup (A_{3i} \cap A_{4i})\right) \cap (A_5 \cup A_6) \\ A_{1i} &= \left\{Y : (\sigma_i V_{i+1,j} - \sigma_{i+1}V_{ij})Y \geqslant \tau_j^2(\sigma_i Z_{i+1,j} - \sigma_{i+1}Z_{ij})\right\}, \\ A_{2i} &= \left\{Y : (\sigma_i V_{i+1,j} + \sigma_{i+1}V_{ij})Y \leqslant \tau_j^2(\sigma_{i+1}Z_{ij} + \sigma_i Z_{i+1,j})\right\}, \\ A_{3i} &= \left\{Y : (\sigma_i V_{i+1,j} + \sigma_{i+1}V_{ij})Y \geqslant \tau_j^2(\sigma_{i+1}Z_{ij} + \sigma_i Z_{i+1,j})\right\}, \\ A_{4i} &= \left\{Y : (\sigma_i V_{i+1,j} - \sigma_{i+1}V_{ij})Y \leqslant \tau_j^2(\sigma_i Z_{i+1,j} - \sigma_{i+1}Z_{ij})\right\}, \\ A_5 &= \left\{Y : V_{Kj}Y \leqslant \tau_j^2\left[Z_K - \sigma_K \Phi^{-1}(1 - p_{\text{crit}}/2)\right]\right\}, \\ A_6 &= \left\{Y : V_{Kj}Y \geqslant \tau_j^2\left[Z_K + \sigma_K \Phi^{-1}(1 - p_{\text{crit}}/2)\right]\right\} \end{aligned}$$

and we define $\min\{\emptyset\} = +\infty$ and $\max\{\emptyset\} = -\infty$.

Proof.

As before, we need to determine how the support of Y changes.

Conditioning on the event Q_2 means that $\frac{|X_i|}{\sigma_i} \geqslant \frac{|X_{i+1}|}{\sigma_{i+1}}$ for $i = 1, \ldots, K-1$. Using equations (1.1), this gives the following

$$\frac{|X_i|}{\sigma_i} \geqslant \frac{|X_{i+1}|}{\sigma_{i+1}} \implies \sigma_{i+1}\left|Z_{ij} - \frac{V_{ij}}{\tau_j^2}Y_j\right| \geqslant \sigma_i\left|Z_{i+1,j} - \frac{V_{i+1,j}}{\tau_j^2}Y_j\right|.$$

There are two initial cases - either

I. $\sigma_{i+1}\left(Z_{ij} - \frac{V_{ij}}{\tau_j^2}Y_j\right) \geqslant \sigma_i\left|Z_{i+1,j} - \frac{V_{i+1,j}}{\tau_j^2}Y_j\right|$, **or**

II. $\sigma_{i+1}\left(Z_{ij} - \frac{V_{ij}}{\tau_j^2}Y_j\right) \leqslant -\sigma_i\left|Z_{i+1,j} - \frac{V_{i+1,j}}{\tau_j^2}Y_j\right|$.

Case I implies that

$$\sigma_i\left(Z_{i+1,j} - \frac{V_{i+1,j}}{\tau_j^2}Y_j\right) \leqslant \sigma_{i+1}\left(Z_{ij} - \frac{V_{ij}}{\tau_j^2}Y_j\right) \qquad \text{and}$$
$$\sigma_i\left(Z_{i+1,j} - \frac{V_{i+1,j}}{\tau_j^2}Y_j\right) \geqslant -\sigma_{i+1}\left(Z_{ij} - \frac{V_{ij}}{\tau_j^2}Y_j\right).$$

These equations correspond to the sets A_{1i} and A_{2i} respectively.

Case II implies that

$$\sigma_i\left(Z_{i+1,j} - \frac{V_{i+1,j}}{\tau_j^2}Y_j\right) \leqslant -\sigma_{i+1}\left(Z_{ij} - \frac{V_{ij}}{\tau_j^2}Y_j\right) \qquad \text{and}$$
$$\sigma_i\left(Z_{i+1,j} - \frac{V_{i+1,j}}{\tau_j^2}Y_j\right) \geqslant \sigma_{i+1}\left(Z_{ij} - \frac{V_{ij}}{\tau_j^2}Y_j\right).$$

These equations correspond to the sets A_{3i} and A_{4i} respectively.

Finally, for $i = K$ we have

$$\frac{|X_K|}{\sigma_K} \geqslant \Phi^{-1}(1 - p_{\text{crit}}/2) \implies \left|Z_{Kj} - \frac{V_{Kj}}{\tau_j^2}Y_j\right| \geqslant \sigma_K\Phi^{-1}(1 - p_{\text{crit}}/2).$$

Again this gives two cases:

I. $Z_{Kj} - \frac{V_{Kj}}{\tau_j^2}Y_j \geqslant \sigma_K\Phi^{-1}(1 - p_{\text{crit}}/2)$, **or**

II. $Z_{Kj} - \frac{V_{Kj}}{\tau_j^2}Y_j \leqslant -\sigma_K\Phi^{-1}(1 - p_{\text{crit}}/2)$.

Case I implies that $V_{Kj}Y_j \leqslant \tau_j^2\left[Z_{Kj} - \sigma_K\Phi^{-1}(1 - p_{\text{crit}}/2)\right]$, corresponding to the set A_5. Case II implies that $V_{Kj}Y_j \geqslant \tau_j^2\left[Z_{Kj} + \sigma_K\Phi^{-1}(1 - p_{\text{crit}}/2)\right]$, corresponding to set A_6.

□

6. Comparison with the estimators of Bowden and Dudbridge (2009)

If we set the off-diagonal terms of the covariance matrix equal to 0, the sufficient, complete statistic Z_{ij} becomes

$$Z_{ij} = \begin{cases} X_j + \frac{\sigma_j^2}{\tau_j^2} Y_j & i = j \\ X_i & i \neq j \end{cases}$$

6.1 *One-sided test*

Starting with the one-sided test, Bowden and Dudbridge (2009) derived the following estimator for μ_j conditional on Q_1:

$$\tilde{\mu}_j = \frac{\tau_j^2 X_j + \sigma_j^2 Y_j}{\sigma_j^2 + \tau_j^2} - \frac{\tau_j^2}{\sqrt{\sigma_j^2 + \tau_j^2}} \frac{\phi(W_{j,j+1}) - \phi(W_{j,j-1})}{\Phi(W_{j,j+1}) - \Phi(W_{j,j-1})}$$

where

$$W_{j,s} = \frac{\sqrt{\sigma_j^2 + \tau_j^2}}{\sigma_j^2} \left(\frac{\tau_j^2 X_j + \sigma_j^2 Y_j}{\sigma_j^2 + \tau_j^2} - \frac{\sigma_j X_s}{\sigma_s} \right).$$

Note that by definition, $W_{1,0} = -\infty$, and $X_{K+1}/\sigma_{K+1} = \Phi^{-1}(1 - p_{\text{crit}})$.

As for our estimator, firstly note that $V_{ij} = 0$ for $i \neq j$, meaning that $\sigma_{i+1} V_{ij} = \sigma_i V_{i+1,j} = 0$ for $i \notin \{j, j-1\}$. Hence the set A_3 only has the term corresponding to $i = j - 1$ (for $j \neq 1$).

Considering the case when $1 < j < K$, then the set A_1 only has the term corresponding to $i = j$, while the sets A_2 and A_4 are empty. This implies that

$$k_1 = \frac{\tau_j^2 (\sigma_{j+1} Z_{jj} - \sigma_j X_{j+1})}{\sigma_{j+1} \sigma_j^2}, \quad k_2 = \frac{\tau_j^2 (\sigma_{j-1} Z_{jj} - \sigma_j X_{j-1})}{\sigma_{j-1} \sigma_j^2}$$

which gives

$$\begin{aligned} W_1 &= \frac{k_1 \sqrt{\sigma_j^2 + \tau_j^2}}{\tau_j^2} - \frac{Z_{jj}}{\sqrt{\sigma_j^2 + \tau_j^2}} \\ &= \frac{\sqrt{\sigma_j^2 + \tau_j^2}}{\sigma_j^2} \left(Z_{jj} - \frac{\sigma_j}{\sigma_{j+1}} X_{j+1} - \frac{\sigma_j^2 Z_{jj}}{\sigma_j^2 + \tau_j^2} \right) \\ &= \frac{\sqrt{\sigma_j^2 + \tau_j^2}}{\sigma_j^2} \left(\frac{\tau_j^2 X_j + \sigma_j^2 Y_j}{\sigma_j^2 + \tau_j^2} - \frac{\sigma_j}{\sigma_{j+1}} X_{j+1} \right) \end{aligned}$$

and similarly

$$W_2 = \frac{\sqrt{\sigma_j^2 + \tau_j^2}}{\sigma_j^2} \left(\frac{\tau_j^2 X_j + \sigma_j^2 Y_j}{\sigma_j^2 + \tau_j^2} - \frac{\sigma_j}{\sigma_{j-1}} X_{j-1} \right).$$

If $j = 1$, then the set A_3 is now also empty, so by definition $k_2 = -\infty$ and hence $W_2 = -\infty$. This agrees with the definition of $W_{1,0} = -\infty$.

Finally, if $j = K$ then the set A_1 is now empty, whereas A_2 is not. This means that $k_1 = \frac{\tau_K^2}{\sigma_K^2}\left[Z_{KK} - \sigma_K \Phi^{-1}(1-p_{\text{crit}})\right]$, which agrees with defining $X_{K+1}/\sigma_{K+1} = \Phi^{-1}(1-p_{\text{crit}})$.

Hence our estimator

$$\hat{U}_j = \frac{\tau_j^2 Z_{jj}}{\sigma_j^2+\tau_j^2} - \frac{\tau_j^2}{\sqrt{\sigma_j^2+\tau_j^2}} \frac{\phi(W_1)-\phi(W_2)}{\Phi(W_1)-\Phi(W_2)}$$

is the same as Bowden and Dudbridge's for all $j \in \{1, \dots, K\}$.

6.2 *Two-sided test*

The two-sided test is more complex. The Bowden and Dudbridge estimator is as follows

$$\tilde{\mu}_j = \frac{\tau_j^2 X_j + \sigma_j^2 Y_j}{\sigma_j^2+\tau_j^2} - \frac{\tau_j^2}{\sqrt{\sigma_j^2+\tau_j^2}} \frac{\phi(W_{j,j+1}^{(0)}) - \phi(W_{j,j-1}^{(0)}) - \phi(W_{j,j+1}^{(1)}) + \phi(W_{j,j-1}^{(1)})}{\Phi(W_{j,j+1}^{(0)}) - \Phi(W_{j,j-1}^{(0)}) - \Phi(W_{j,j+1}^{(1)}) + \Phi(W_{j,j-1}^{(1)})}$$

where

$$W_{j,s}^{(p)} = \frac{\sqrt{\sigma_j^2+\tau_j^2}}{\sigma_j^2}\left(\frac{\tau_j^2 X_j + \sigma_j^2 Y_j}{\sigma_j^2+\tau_j^2} - (-1)^p \frac{\sigma_j |X_s|}{\sigma_s}\right).$$

Note that by definition, $W_{1,0}^{(0)} = -\infty$, $W_{1,0}^{(1)} = \infty$ and $|X_{K+1}|/\sigma_{K+1} = \Phi^{-1}(1-p_{\text{crit}}/2)$.

As before, since $V_{ij} = 0$ for $i \neq j$, then $\sigma_{i+1}V_{ij} = \sigma_i V_{i+1,j} = 0$ for $i \notin \{j, j-1\}$. Considering first the case when $1 < j < K$, then the sets A_5 and A_6 are empty and the only contributions to the sets A_1, A_2, A_3 and A_4 are as follows:

$$\begin{aligned}
A_{1,j-1} &= \left\{Y : \sigma_j^2\sigma_{j-1}Y \geqslant \tau_j^2(\sigma_{j-1}Z_{jj} - \sigma_j X_{j-1})\right\} && i = j-1\\
A_{1,j} &= \left\{Y : -\sigma_j^2\sigma_{j+1}Y \geqslant \tau_j^2(\sigma_j X_{j+1} - \sigma_{j+1}Z_{jj})\right\} && i = j\\
A_{2,j-1} &= \left\{Y : \sigma_j^2\sigma_{j-1}Y \leqslant \tau_j^2(\sigma_j X_{j-1} + \sigma_{j-1}Z_{jj})\right\} && i = j-1\\
A_{2,j} &= \left\{Y : \sigma_j^2\sigma_{j+1}Y \geqslant \tau_j^2(\sigma_j X_{j+1} + \sigma_{j+1}Z_{jj})\right\} && i = j\\
A_{3,j-1} &= \left\{Y : \sigma_j^2\sigma_{j-1}Y \geqslant \tau_j^2(\sigma_j X_{j-1} + \sigma_{j-1}Z_{jj})\right\} && i = j-1\\
A_{3,j} &= \left\{Y : \sigma_j^2\sigma_{j+1}Y \geqslant \tau_j^2(\sigma_j X_{j+1} + \sigma_{j+1}Z_{jj})\right\} && i = j\\
A_{4,j-1} &= \left\{Y : \sigma_j^2\sigma_{j-1}Y \leqslant \tau_j^2(\sigma_{j-1}Z_{jj} - \sigma_j X_{j-1})\right\} && i = j-1\\
A_{4,j} &= \left\{Y : -\sigma_j^2\sigma_{j+1}Y \leqslant \tau_j^2(\sigma_j X_{j+1} - \sigma_{j+1}Z_{jj})\right\} && i = j
\end{aligned}$$

From above, for $i = j-1$ we either have

$$\frac{\tau_j^2\left(\sigma_{j-1}Z_{jj} - \sigma_j X_{j-1}\right)}{\sigma_{j-1}\sigma_j^2} \leqslant Y \leqslant \frac{\tau_j^2\left(\sigma_{j-1}Z_{jj} + \sigma_j X_{j-1}\right)}{\sigma_{j-1}\sigma_j^2}$$

or

$$\frac{\tau_j^2\left(\sigma_j X_{j-1} + \sigma_{j-1}Z_{jj}\right)}{\sigma_{j-1}\sigma_j^2} \leqslant Y \leqslant \frac{\tau_j^2\left(\sigma_{j-1}Z_{jj} - \sigma_j X_{j-1}\right)}{\sigma_{j-1}\sigma_j^2}.$$

For $i = j$ we either have

$$Y \leqslant \min\left(\frac{\tau_j^2\left(\sigma_j X_{j+1} + \sigma_{j+1}Z_{jj}\right)}{\sigma_{j+1}\sigma_j^2}, \frac{\tau_j^2\left(\sigma_{j+1}Z_{jj} - \sigma_j X_{j+1}\right)}{\sigma_{j+1}\sigma_j^2}\right)$$

or

$$Y \geqslant \max\left(\frac{\tau_j^2\left(\sigma_{j+1}Z_{jj}+\sigma_j X_{j+1}\right)}{\sigma_{j+1}\sigma_j^2}, \frac{\tau_j^2\left(\sigma_{j+1}Z_{jj}-\sigma_j X_{j+1}\right)}{\sigma_{j+1}\sigma_j^2}\right).$$

These equations reduce to $B_1 \leqslant Y \leqslant B_2$ **and** either $Y \leqslant B_3$ **or** $Y \geqslant B_4$ where

$$\begin{aligned} B_1 &= \frac{\tau_j^2\left(\sigma_{j-1}Z_{jj}-\sigma_j|X_{j-1}|\right)}{\sigma_{j-1}\sigma_j^2} = \frac{\tau_j^2}{\sigma_j^2}\left(Z_{jj}-\sigma_j\frac{|X_{j-1}|}{\sigma_{j-1}}\right)\\ B_2 &= \frac{\tau_j^2\left(\sigma_{j-1}Z_{jj}+\sigma|X_{j-1}|\right)}{\sigma_{j-1}\sigma_j^2} = \frac{\tau_j^2}{\sigma_j^2}\left(Z_{jj}+\sigma_j\frac{|X_{j-1}|}{\sigma_{j-1}}\right)\\ B_3 &= \frac{\tau_j^2\left(\sigma_{j+1}Z_{jj}-\sigma_j|X_{j+1}|\right)}{\sigma_{j+1}\sigma_j^2} = \frac{\tau_j^2}{\sigma_j^2}\left(Z_{jj}-\sigma_j\frac{|X_{j+1}|}{\sigma_{j+1}}\right)\\ B_4 &= \frac{\tau_j^2\left(\sigma_{j+1}Z_{jj}+\sigma_j|X_{j+1}|\right)}{\sigma_{j+1}\sigma_j^2} = \frac{\tau_j^2}{\sigma_j^2}\left(Z_{jj}+\sigma_j\frac{|X_{j+1}|}{\sigma_{j+1}}\right). \end{aligned}$$

Noting that $|X_{j-1}|/\sigma_{j-1} \geqslant |X_{j+1}|/\sigma_{j+1}$ we can see that $B_1 \leqslant B_2, B_1 \leqslant B_3, B_1 \leqslant B_4$, $B_2 \geqslant B_3, B_2 \geqslant B_4$ and $B_3 \leqslant B_4$. Thus the inequalities reduce to

$$B_1 = \frac{\tau_j^2}{\sigma_j^2}\left(Z_{jj}-\sigma_j\frac{|X_{j-1}|}{\sigma_{j-1}}\right) \leqslant Y \leqslant \frac{\tau_j^2}{\sigma_j^2}\left(Z_{jj}-\sigma_j\frac{|X_{j+1}|}{\sigma_{j+1}}\right) = B_3$$

and

$$B_4 = \frac{\tau_j^2}{\sigma_j^2}\left(Z_{jj}+\sigma_j\frac{|X_{j+1}|}{\sigma_{j+1}}\right) \leqslant Y \leqslant \frac{\tau_j^2}{\sigma_j^2}\left(Z_{jj}+\sigma_j\frac{|X_{j-1}|}{\sigma_{j-1}}\right) = B_2.$$

Hence $\bigcup_{i=1}^M [a_i, b_i] = [B_1, B_3] \cup [B_4, B_2]$, and we can plug these values into W_{1i} and W_{2i} $(i = 1, 2)$. Going through the algebra (in a very similar way to the one-sided test) gives the same formulae as that by Bowden and Dudbridge.

If $j = 1$, then the sets A_5 and A_6 remain empty, but the contributions to the sets A_1, A_2, A_3 and A_4 from $i = j - 1$ disappear. Hence $B_1 = -\infty$ and $B_2 = \infty$, which agrees with defining $W_{1,0}^{(0)} = -\infty$ and $W_{1,0}^{(1)} = \infty$.

Finally, if $j = K$ then the contributions to the sets A_1, A_2, A_3 and A_4 from $i = j$ disappear. Instead, we now have the following contributions from the sets A_5 and A_6:

$$Y \leqslant \frac{\tau_K^2}{\sigma_K^2}\left[Z_{KK}-\sigma_K\Phi^{-1}(1-p_{\text{crit}}/2)\right] = B_3 \;\; \textbf{or} \;\; Y \geqslant \frac{\tau_K^2}{\sigma_K^2}\left[Z_{KK}+\sigma_K\Phi^{-1}(1-p_{\text{crit}}/2)\right] = B_1.$$

Again, this agrees with the definition of $|X_{K+1}|/\sigma_{K+1} = \Phi^{-1}(1 - p_{\text{crit}}/2)$.

Hence our estimator

$$\hat{U}_j = \frac{\tau_j^2 Z_{jj}}{\sigma_j^2+\tau_j^2} - \frac{\tau_j^2}{\sqrt{\sigma_j^2+\tau_j^2}}\frac{\sum_{i=1}^M \phi(W_{1i})-\phi(W_{2i})}{\sum_{i=1}^M \Phi(W_{1i})-\Phi(W_{2i})}$$

is the same as Bowden and Dudbridge's for all $j \in \{1, \ldots, K\}$.

7. Simulating conditional log odds ratios in the multivariate normal setting

7.1 *Bivariate case*

Consider first simulating X_1 and X_2 from a bivariate normal distribution

$$\begin{pmatrix} X_1 \\ X_2 \end{pmatrix} \sim N\left(\begin{pmatrix} \mu_1 \\ \mu_2 \end{pmatrix}, \begin{pmatrix} \sigma_1^2 & \rho\sigma_1\sigma_2 \\ \rho\sigma_1\sigma_2 & \sigma_2^2 \end{pmatrix}\right),$$

conditional on $q_1^U \geqslant |X_1|/\sigma_1 \geqslant q_1^L$ and $q_2^U \geqslant |X_2|/\sigma_2 \geqslant q_2^L$.

Define

$$\pi_i^L = \Phi(-q_i^L - \mu_i/\sigma_i) - \Phi(-q_i^U - \mu_i/\sigma_i)$$
$$\pi_i^R = \Phi(q_i^U - \mu_i/\sigma_i) - \Phi(q_i^L - \mu_i/\sigma_i)$$

for $i = 1, 2$. Then π_i^L and π_i^R give the left- and right-sided power respectively to detect an effect size μ_i with variance σ_i^2.

We get a bootstrap sample $\left(X_1^{(B)}, X_2^{(B)}\right)$ from the above bivariate normal distribution for (X_1, X_2), truncated so that $a_1 < X_1 < b_1$ and $a_2 < X_2 < b_2$ (where a_1, a_2, b_1 and b_2 are defined below). To sample from such a truncated bivariate normal distribution, we use the R package `tmvtnorm`.

The truncation boundaries a_1, a_2, b_1 and b_2 are chosen as follows. Independently for $i = 1, 2$, with probability $\frac{\pi_i^R}{\pi_i^R+\pi_i^L}$ we set $a_i = q_i^L - \mu_i/\sigma_i$ and $b_i = q_i^U - \mu_i/\sigma_i$, while with probability $\frac{\pi_i^L}{\pi_i^R+\pi_i^L}$ we set $a_i = -q_i^U - \mu_i/\sigma_i$ and $b_i = -q_i^L - \mu_i/\sigma_i$.

Now consider simulating X_1 and X_2 from a bivariate normal distribution

$$\begin{pmatrix} X_1 \\ X_2 \end{pmatrix} \sim N\left(\begin{pmatrix} \mu_1 \\ \mu_2 \end{pmatrix}, \begin{pmatrix} \sigma_1^2 & \rho\sigma_1\sigma_2 \\ \rho\sigma_1\sigma_2 & \sigma_2^2 \end{pmatrix}\right),$$

conditional on $|X_1|/\sigma_1 \geqslant |X_2|/\sigma_2 \geqslant q_2^L$.

As before, we define π_i^L and π_i^R, where this time we set $q_1^U = q_2^U = \infty$ and $q_1^L = q_2^L$. This gives $\pi_i^L = \Phi(-q_2^L - \mu_i/\sigma_i)$ and $\pi_i^R = 1 - \Phi(q_2^L - \mu_i/\sigma_i)$ for $i = 1, 2$.

We first get a bootstrap sample $X_2^{(B)}$, where $X_2^{(B)} \sim N(\mu_2, \sigma_2^2)$ and is truncated so that $a_2 < X_2^{(B)} < b_2$. Using this value of $X_2^{(B)}$, we then can get a bootstrap sample $X_1^{(B)}$, where $X_1^{(B)} \sim N\left(\mu_1 + \frac{\sigma_1}{\sigma_2}\rho\left(X_2^{(B)} - \mu_2\right), (1-\rho^2)\sigma_1^2\right)$ and is truncated so that $a_1 < X_1^{(B)} < b_1$ (where a_1, a_2, b_1 and b_2 are defined below). To simulate from the truncated (univariate) normal distributions above, we use the R package `truncnorm`. The normal distribution for $X_1^{(B)}$ has been chosen because $X_1|X_2 = x_2 \sim N\left(\mu_1 + \rho\frac{\sigma_1}{\sigma_2}(x_2 - \mu_2), (1-\rho^2)\sigma_1^2\right)$.

The truncation boundaries a_1, a_2, b_1 and b_2 are chosen as follows. With probability $\frac{\pi_2^R}{\pi_2^R+\pi_2^L}$ we set $a_2 = \sigma_2 q_2^L$ and $b_2 = \infty$, while with probability $\frac{\pi_2^L}{\pi_2^R+\pi_2^L}$ we set $a_2 = -\infty$ and $b_2 = -\sigma_2 q_2^L$. Then, with probability $\frac{\pi_1^R}{\pi_1^R+\pi_1^L}$ we set $a_1 = \sigma_1|X_2^{(B)}|/\sigma_2$ and $b_1 = \infty$, while with probability $\frac{\pi_1^L}{\pi_1^R+\pi_1^L}$ we set $a_1 = -\infty$ and $b_1 = -\sigma_1|X_2^{(B)}|/\sigma_2$

7.2 *Extension to the general multivariate normal case*

Consider simulating $\boldsymbol{X} = (X_1, \ldots, X_K)$ from a multivariate normal distribution $\boldsymbol{X} \sim N(\boldsymbol{\mu}, V)$, conditional on $q_i^U \geqslant |X_i|/\sigma_i \geqslant q_i^L$ for $i = 1, \ldots, K$ (where $\sigma_i^2 = V_{ii}$).

As before, for $i = 1, \dots, K$ we define

$$\pi_i^L = \Phi(-q_i^L - \mu_i/\sigma_i) - \Phi(-q_i^U - \mu_i/\sigma_i)$$
$$\pi_i^R = \Phi(q_i^U - \mu_i/\sigma_i) - \Phi(q_i^L - \mu_i/\sigma_i)$$

We then get a bootstrap sample $\left(X_i^{(B)}\right)_{i=1,\dots,K}$ from the multivariate normal distribution $N(\boldsymbol{\mu}, V)$, truncated so that $a_i < X_i^{(B)} < b_i$ for $i = 1, \dots, K$ (where the a_i and b_i are defined below). To sample from such a truncated multivariate normal distribution, we can use the R package `tmvtnorm`.

The truncation boundaries a_i and b_i are chosen as follows. Independently for $i = 1, \dots, K$, with probability $\frac{\pi_i^R}{\pi_i^R + \pi_i^L}$ we set $a_i = q_i^L - \mu_i/\sigma_i$ and $b_i = q_i^U - \mu_i/\sigma_i$, while with probability $\frac{\pi_i^L}{\pi_i^R + \pi_i^L}$ we set $a_i = -q_i^U - \mu_i/\sigma_i$ and $b_i = -q_i^L - \mu_i/\sigma_i$.

Now consider the case where we want to simulate $\boldsymbol{X} = (X_1, \dots, X_K)$ from a multivariate normal distribution $\boldsymbol{X} \sim N(\boldsymbol{\mu}, V)$, conditional on $|X_1|/\sigma_1 \geqslant \dots \geqslant |X_K|/\sigma_K \geqslant q^L$.

As before, we set $\pi_i^L = \Phi(-q^L - \mu_i/\sigma_i)$ and $\pi_i^R = 1 - \Phi(q^L - \mu_i/\sigma_i)$ for $i = 1, \dots, K$. To simulate our bootstrap samples, we use the following result:

Lemma 7.1 Suppose $\boldsymbol{X} = (X_1, \dots, X_K)$ follows a multivariate normal distribution $\boldsymbol{X} \sim N(\boldsymbol{\mu}, V)$. Then conditional on $X_K = x$, the distribution of $(X_1, \dots, X_{K-1})$ is multivariate normal $N(\tilde{\boldsymbol{\mu}}, \widetilde{V})$, where $\tilde{\mu}_i = \mu_i + V_{iK}(x - \mu_K)/\sigma_K^2$ and $\widetilde{V}_{ij} = V_{ij} - V_{iK}V_{jK}/\sigma_K^2$ for $i, j \in \{1, \dots, K-1\}$.

Hence we can proceed inductively in K. We have already shown how to simulate $\boldsymbol{X}$ when $K = 2$, so let us consider the case when $K = 3$. Firstly, using the R package `truncnorm`, we get a bootstrap sample $X_3^{(B)} \sim N(\mu_3, \sigma_3^2)$, truncated so that $a_3 < X_3^{(B)} < b_3$.

The truncation boundaries a_3 and b_3 are chosen as follows. With probability $\frac{\pi_3^R}{\pi_3^R + \pi_3^L}$ we set $a_3 = \sigma_3 q^L$ and $b_3 = \infty$, while with probability $\frac{\pi_3^L}{\pi_3^R + \pi_3^L}$ we set $a_3 = -\infty$ and $b_3 = -\sigma_3 q^L$.

Conditional on $X_3 = X_3^{(B)}$, (X_1, X_2) follows the conditional multivariate normal distribution given in Lemma 7.1, which is actually bivariate normal since $K = 3$. We now need to simulate (X_1, X_2) conditional on $|X_2|/\sigma_1 \geqslant |X_2|/\sigma_2 \geqslant |X_3^{(B)}|/\sigma_3$, which is just the bivariate case with mean $\tilde{\boldsymbol{\mu}}$, covariance matrix $\widetilde{V}$ and lower truncation boundary $q_2^L = |X_3^{(B)}|/\sigma_3$.

Hence we can simulate $\boldsymbol{X}$ when $K = 3$. Continuing inductively and repeatedly using Lemma 7.1, we can thus simulate $\boldsymbol{X}$ for all $K > 2$.

References

Bowden, J. and Dudbridge, F. (2009). Unbiased estimation of odds ratios: combining genomewide association scans with replication studies. *Genetic Epidemiology* **33**(5), 406–418.

Bowden, J. and Glimm, E. (2008). Unbiased estimation of selected treatment means in two-stage trials. *Biometrical Journal* **50**(4), 515–527.

Lehmann, E. L. and Romano, J. P. (2005). *Testing statistical hypotheses*. Springer.

[*Received June 23, 2015; revised November 11, 2015; accepted for publication January 15, 2016*]
